# Cardiac-specific ITCH overexpression ameliorates septic cardiomyopathy *via* inhibition of the NF-κB signaling pathway

**DOI:** 10.1016/j.jmccpl.2022.100018

**Published:** 2022-11-02

**Authors:** Yuji Saito, Yoichiro Otaki, Tetsu Watanabe, Shingo Tachibana, Junya Sato, Yuta Kobayashi, Tomonori Aono, Jun Goto, Masahiro Wanezaki, Daisuke Kutsuzawa, Shigehiko Kato, Harutoshi Tamura, Satoshi Nishiyama, Takanori Arimoto, Hiroki Takahashi, Masafumi Watanabe

**Affiliations:** Department of Cardiology, Pulmonology and Nephrology, Yamagata University School of Medicine, 2-2-2 Iida-Nishi, Yamagata 990-9585, Japan

**Keywords:** ITCH, NF-κB, Ubiquitylation, Septic cardiomyopathy

## Abstract

**Background:**

Septic cardiomyopathy is a common complication of septic shock and organ dysfunction. ITCH is a HECT (homologous to the E6-AP carboxyl-terminus)-type ubiquitin E3 ligase that plays a critical role in inflammatory suppression. Herein, we focused on the interaction between ITCH and key regulators of nuclear factor-κB (NF-κB), such as tumor necrosis factor receptor-associated factor 6 (TRAF6) and transforming growth factor-β activated kinase 1 (TAK1), and examined the impact of ITCH on the development of septic cardiomyopathy.

**Methods and results:**

In H9C2 cardiomyocytes, ITCH protein expression decreased in response to lipopolysaccharide (LPS) and tumor necrosis factor alpha (TNFα). The protein interactions of ITCH with TRAF6 and TAK1 were confirmed by immunoprecipitation *in vitro* and *in vivo*. Based on overexpression and knockdown studies of ITCH in H9C2 cardiomyocytes, ITCH regulates the phosphorylation of NF-κB and subsequent interleukin 6 (*IL-6*) expression in response to LPS and TNFα stimulation. LPS was intraperitoneally injected into transgenic mice with cardiac-specific overexpression of ITCH (ITCH-Tg) and wild-type (WT) mice. Compared with WT mice, phosphorylation of NF-κB and subsequent *IL-6* expression were inhibited in ITCH-Tg mice. Cardiac systolic dysfunction after LPS administration was ameliorated in ITCH-Tg mice, and the survival rate was higher in ITCH-Tg mice than in WT mice.

**Conclusion:**

ITCH interacts with TRAF6 and TAK1 in cardiomyocytes and improves cardiac function and survival rates in septic cardiomyopathy by suppressing the NF-κB pathway.

## Highlights

• ITCH interacted with TRAF6 and TAK1 in cardiomyocytes.
• ITCH modulates activation of NFκB in cardiomyocytes.
• Cardiac overexpression of ITCH preserves cardiac function in septic cardiomyopathy.
• Cardiac overexpression of ITCH improves survival rate in septic cardiomyopathy.
• ITCH plays a protective role in the development of septic cardiomyopathy.

## Graphical abstract

**Figure:**
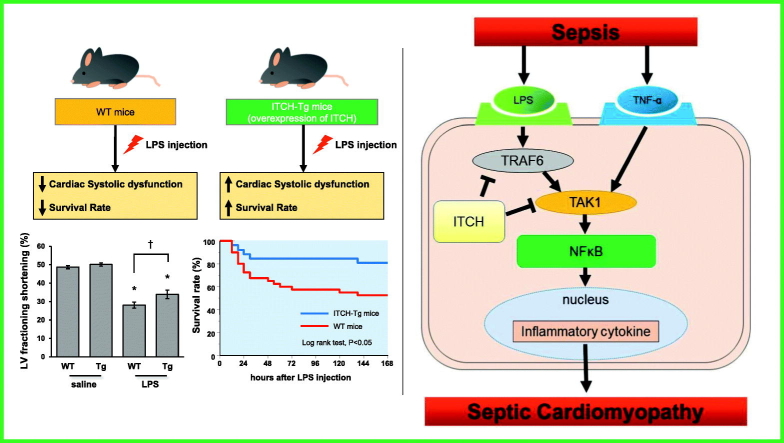

## 1 Introduction

Despite advances in medicine, sepsis remains an increasing public health problem associated with a high mortality rate [1]. Sepsis has recently been redefined as a life-threatening organ dysfunction caused by a dysregulated host response to infection, highlighting the central role of organ dysfunction in the pathogenesis of sepsis and as a determinant of poor outcomes [1]. Septic cardiomyopathy is a common complication, estimated to occur in 10 to 70 % of patients with sepsis, and is responsible for not only septic shock but also organ dysfunction, complicating the therapeutic management of patients with sepsis [2], [3], [4]. The myocardium is functionally and structurally damaged in patients with septic cardiomyopathy. However, the mechanism and treatment of septic cardiomyopathy remain poorly clarified.

The current understanding is that septic cardiomyopathy is induced by pathogen-associated molecular patterns, including endotoxin, cytokines, damage-associated molecular patterns, and complements [5], [6]. Typically, both innate and adaptive immunity are involved in mediating the response to tissue injury, owing to pathogen-associated molecular patterns through nuclear factor-κB (NF-κB) activation and subsequent pro-inflammatory cytokine production in the heart [7], indicating that NF-κB activation in immune cells is key to the development of septic cardiomyopathy [8]. In contrast, accumulating evidence has demonstrated that NF-κB signaling in the myocardium is also involved in septic cardiomyopathy. Thus, NF-κB signaling in both immune cells and cardiomyocytes is a potential therapeutic target in septic cardiomyopathy. Although NF-κB signaling is regulated by ubiquitylation [9], the role of ubiquitylation in septic cardiomyopathy has not been examined.

Ubiquitylation and deubiquitylation are post-translational modifications that play an important role in regulating cardiovascular homeostasis [10]. HECT (homologous to the E6-AP carboxyl-terminus)-type ubiquitin E3 ligase ITCH is a 113-kDa protein with an N-terminal C2 domain, four WW domains, and a HECT domain [11], [12]. ITCH reportedly interacts with key regulators of NF-κB signaling, such as tumor necrosis factor receptor-associated factor 6 (TRAF6) and transforming growth factor-β activated kinase 1 (TAK1) [13], [14]. Importantly, it has been reported that ITCH deficiency in immune cells leads to persistent activation of NF-κB signaling, resulting in inflammation [15], [16], [17]. Additionally, it has been reported to regulate the interplay between innate and adaptive immune cells [18]; however, the role of ITCH in the myocardium has not been comprehensively examined in the development of septic cardiomyopathy.

We hypothesized that ITCH interacts with TRAF6 and TAK1 in cardiomyocytes and prevents the development of septic cardiomyopathy by inhibiting the NF-κB signaling pathway. However, it remains unclear whether the development of septic cardiomyopathy is associated with insufficient ITCH-dependent inhibition of the NF-κB signaling pathway in response to lipopolysaccharide (LPS) and tumor necrosis factor alpha (TNFα) stimulation. To clarify the potential role of ITCH in the development of septic cardiomyopathy, we performed ITCH overexpression and knockdown studies in cardiomyocytes and induced septic cardiomyopathy in cardiac-specific ITCH transgenic mice using LPS and TNFα. The purpose of the present study was to examine (1) whether ITCH suppresses NF-κB signaling in cardiomyocytes and (2) whether cardiac function and survival rate are improved in ITCH transgenic mice after intraperitoneal LPS stimulation.

## 2 Methods

The authors declare that all supporting data are available in the article and in the relevant online supplement. Experimental animals were handled according to the animal welfare regulations of Yamagata University, and the Animal Subjects Committee of Yamagata University approved the study protocol (#R3035). This study was conducted in accordance with the Guide for the Care and Use of Laboratory Animals published by the US National Institutes of Health.

Briefly, H9C2 cells were cultured and treated with LPS (0.3 μg/μL) and TNFα (20 ng/mL) [19]. Overexpression and knockdown of ITCH were performed using plasmid and siRNA transfection, respectively. C57BL/6J wild-type (WT) and cardiac-specific ITCH-overexpressing transgenic (ITCH-Tg) mice were administered LPS intraperitoneally to induce septic cardiomyopathy [20], [21]. Immunoprecipitation was performed to examine the protein interaction between ITCH and key components of the NF-κB signaling pathway. Following intraperitoneal LPS injection, cardiac function was assessed by transthoracic echocardiography using Vevo2100 ultrasound echocardiography (Primetech Corporation, Tokyo, Japan). A detailed and expanded methodology is described in the online supplement section.

### 2.1 Statistical analysis

Data values are expressed as mean ± standard error of the mean (SEM) in the figures. Continuous variables were analyzed using Student's *t*-test. Statistical differences among the four groups were determined using Tukey-Kramer analysis. Survival curves after LPS injection were constructed using the Kaplan-Meier method and compared using the log-rank test. Statistical significance was set at a *P*-value < 0.05. All statistical analyses were performed using a standard software package (JMP version 14; SAS Institute, Cary, NC, USA).

## 3 Results

### 3.1 *In vitro* study

#### 3.1.1 ITCH expression after LPS and TNFα stimulation in H9C2 cells

**Fig. 1:**
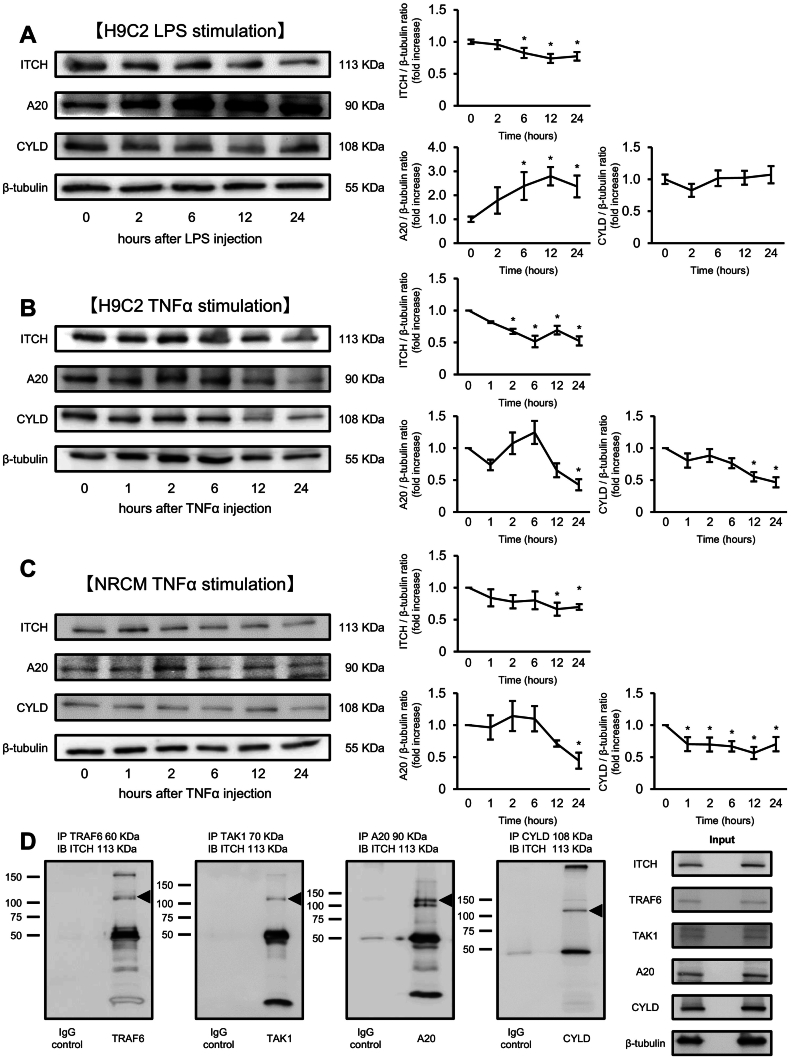
Protein expression levels of ITCH serially decrease following LPS or TNFα stimulation. (A) Representative western blot analysis of ITCH/A20/CYLD in H9C2 cells following LPS stimulation (0.3 μg/μL). Quantification of ITCH/A20/CYLD levels in H9C2 cells following LPS stimulation (*n* = 4 per group). (B) Representative western blot analysis of ITCH/A20/CYLD in H9C2 cells following TNFα stimulation (20 ng/mL). Quantification of ITCH/A20/CYLD levels in H9C2 cells following TNFα stimulation (n = 4 per group). (C) Representative western blot analysis of ITCH/A20/CYLD in neonatal rat cardiomyocytes following TNFα stimulation (20 ng/mL). Quantification of these proteins in neonatal rat cardiomyocytes following TNFα stimulation (n = 4 per group). (D) Immunoprecipitation showing that ITCH interacts with TRAF6/TAK1/A20/CYLD endogenously. Western blots with ITCH antibody after immunoprecipitation show interactions of ITCH with these proteins in H9C2 cell lysates. Data are expressed as mean ± standard error of the mean (SEM) (*P < 0.05 *vs.* control H9C2 cells by Tukey-Kramer analysis). LPS, lipopolysaccharide; NRCM, neonatal rat cardiomyocytes; TAK1, transforming growth factor-β activated kinase 1; TNFα, tumor necrosis factor alpha; TRAF6, tumor necrosis factor receptor-associated factor 6.

Given that LPS and TNFα play a causal role in the development of septic cardiomyopathy [21], [22], we stimulated cardiomyocytes with LPS or TNFα. It remains unclear whether the expression levels of ITCH protein are altered post cardiomyocyte stimulation. Accordingly, we examined serial changes in the endogenous expression levels of ITCH in response to LPS or TNFα. As shown in Fig. 1A and B, the protein expression levels of ITCH were serially decreased after LPS and TNFα stimulation, respectively. A previous report has described that ITCH modulates the deubiquitylation of regulators of the NF-κB signaling pathway by cooperating with ubiquitin-editing enzyme A20 and deubiquitylation enzyme CYLD [23]. Therefore, we also examined serial changes in the endogenous expression levels of A20 and CYLD in response to LPS and TNFα. As shown in Fig. 1A, the protein expression levels of A20 were increased serially after LPS stimulation. In contrast, the protein expression levels of CYLD were serially decreased after TNFα stimulation (Fig. 1B). We also examined serial changes in the endogenous expression levels of ITCH, A20 and CYLD in neonatal rat cardiomyocytes after TNFα stimulation. As shown in Fig. 1C, the protein expression levels of these proteins were also serially decreased after TNFα stimulation.

To examine the interaction between ITCH and key regulators of the NF-κB pathway, we performed immunoprecipitation in H9C2 cells endogenously. As shown in Fig. 1D, ITCH interacted with TRAF6 and TAK1. No previous report has examined whether ITCH can bind to the ubiquitin-editing enzyme A20 and the deubiquitylation enzyme CYLD in cardiomyocytes. Immunoprecipitation showed that ITCH also interacted with A20 and CYLD in H9C2 cells endogenously (Fig. 1D). Additionally, we confirmed the interaction between ITCH and these proteins in ITCH-overexpressing H9C2 cells (Fig. S1).

#### 3.1.2 Overexpression of ITCH inhibited the NF-κB signaling pathway in H9C2 cells

**Fig. 2:**
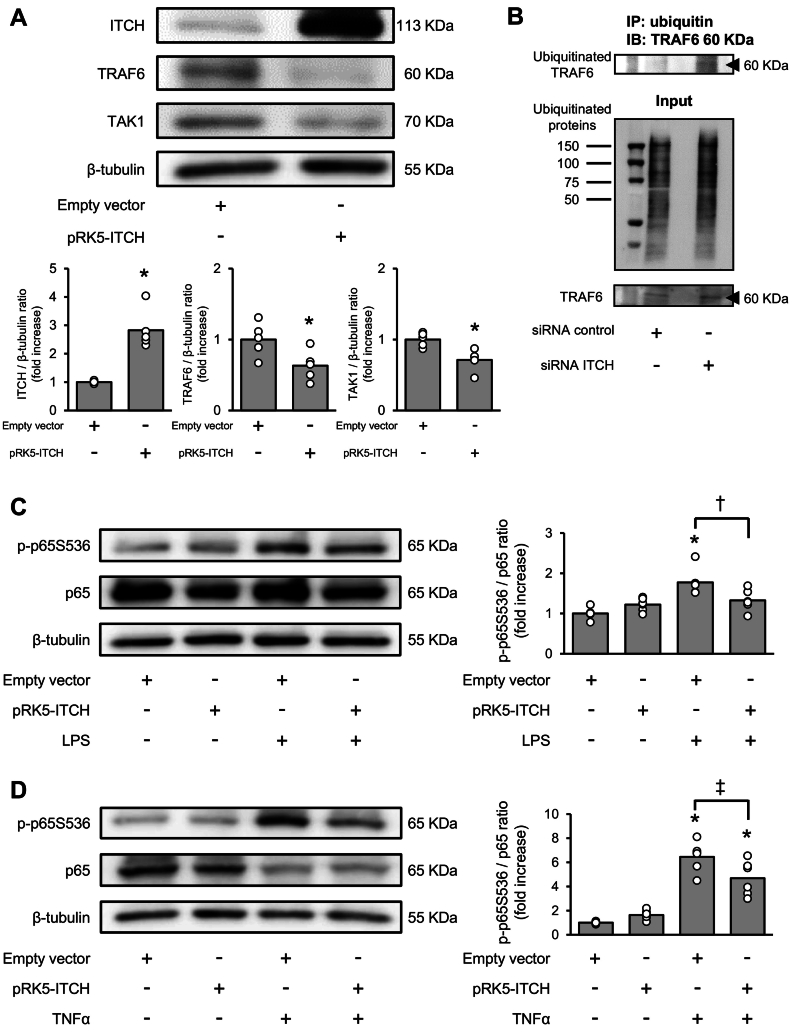
Overexpression of ITCH suppresses NF-κB signaling pathway in H9C2 cells. (A) Representative western blot analysis of ITCH/TRAF6/TAK1 after ITCH overexpression. Quantification of ITCH, TRAF6, and TAK1 levels after ITCH overexpression (*n* = 5–6 per group). (B) Knockdown of ITCH increases ubiquitinated TRAF6 protein, as shown by immunoprecipitation. (C) Representative western blot image of phospho-p65 and p65 in empty vector-transfected and ITCH-overexpressing H9C2 cells 24 h after LPS stimulation (0.3 μg/μL). Quantification of phospho- p65 protein levels in empty vector-transfected and ITCH-overexpressing H9C2 cells 24 h after LPS stimulation (*n* = 6 per group). (D) Representative western blot image of phospho-p65 and p65 in empty vector-transfected and ITCH-overexpressing H9C2 cells 15 min after TNFα stimulation (20 ng/mL). Quantification of phospho-p65 protein levels in empty vector-transfected and ITCH-overexpressing H9C2 cells 15 min after TNFα stimulation (*n* = 6 per group). Data are expressed as mean ± standard error of the mean (SEM) (*P < 0.05 *vs.* empty vector-transfected H9C2 cells, †P < 0.05 *vs.* empty vector-transfected H9C2 cells after LPS stimulation, ‡P < 0.05 *vs.* empty vector-transfected H9C2 cells after TNFα stimulation by Student's *t*-test or Tukey-Kramer analysis). LPS, lipopolysaccharide; TAK1, transforming growth factor-β activated kinase 1; TNFα, tumor necrosis factor alpha; TRAF6, tumor necrosis factor receptor-associated factor 6.

To examine the effect of ITCH on the protein expression levels of TRAF6 and TAK1, we performed a western blot analysis. Protein expression levels of TRAF6 and TAK1 were significantly lower in ITCH-overexpressing H9C2 cells than in H9C2 cells transfected with the empty vector (Fig. 2A). Next, we selectively isolated the ubiquitinated proteins and performed immunoprecipitation. As shown in Fig. 2B, the ubiquitinated TRAF6 protein was augmented in ITCH-Knockdown H9C2 cells. To examine whether overexpression of ITCH inhibits NF-κB signaling in cardiomyocytes, H9C2 cells were stimulated with LPS or TNFα following ITCH overexpression. As shown in Fig. 2C and D, phosphorylation levels of p65 were significantly lower in ITCH-overexpressing H9C2 cells than in H9C2 cells transfected with the empty vector. These findings suggested that ITCH modulates the protein expression levels of key regulators of NF-κB signaling.

#### 3.1.3 Knockdown of ITCH exacerbated NF-κB signaling activation in H9C2 cells

**Fig. 3:**
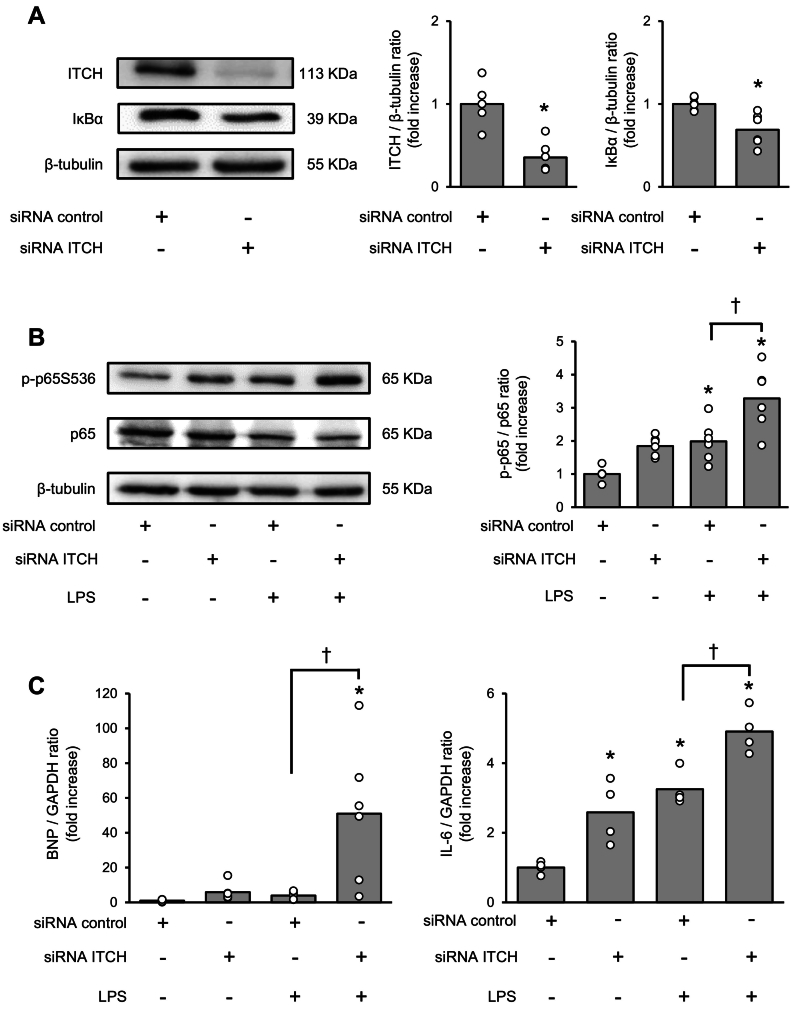
Knockdown of ITCH augments NF-κB signaling pathway in H9C2 cells after LPS stimulation. (A) Expression levels of ITCH and IκBα in siRNA-control- or siRNA-ITCH-transfected H9C2 cells, as determined by western blot analysis (*n* = 4–6 per group). (B) Representative western blot analysis of phospho-p65 in siRNA-control- or siRNA-ITCH-transfected H9C2 cells 24 h after LPS stimulation (0.3 μg/μL). Quantification of phospho-p65 protein levels in siRNA-control- or siRNA-ITCH-transfected H9C2 cells 24 h after LPS stimulation (*n* = 6 per group). (C) Quantitative analysis of *Bnp* and *IL-6* gene expression in siRNA-control- or siRNA-ITCH-transfected H9C2 cells treated with LPS. (*Bnp*: 0.3 μg/μL, 24 h stimulation, n = 6 per group; *IL-6*: 0.3 μg/μL, 6 h stimulation, n = 4 per group). Data are expressed as mean ± standard error of the mean (SEM) (*P < 0.05 *vs.* siRNA-control-transfected H9C2 cells, †P < 0.05 *vs.* siRNA-control-transfected H9C2 cells after LPS stimulation by Student's *t*-test or Tukey-Kramer analysis). IL-6, interleukin-6; LPS, lipopolysaccharide; TNFα, tumor necrosis factor alpha; TRAF6, tumor necrosis factor receptor-associated factor 6.

To examine whether ITCH knockdown exacerbates NF-κB signaling *in vitro*, H9C2 cells were stimulated with LPS following ITCH knockdown. The protein expression levels of IκBα were significantly lower in ITCH-knockdown H9C2 cells than in control-siRNA-transfected H9C2 cells (Fig. 3A). As shown in Fig. 3B, the p65 phosphorylation level was significantly higher in siRNA-ITCH-transfected H9C2 cells than in control-siRNA-transfected H9C2 cells following LPS stimulation. Following ITCH knockdown in LPS-treated H9C2 cells, mRNA expression levels of *Bnp* and interleukin 6 (*IL-6*) were significantly upregulated (Fig. 3C).

**Fig. 4:**
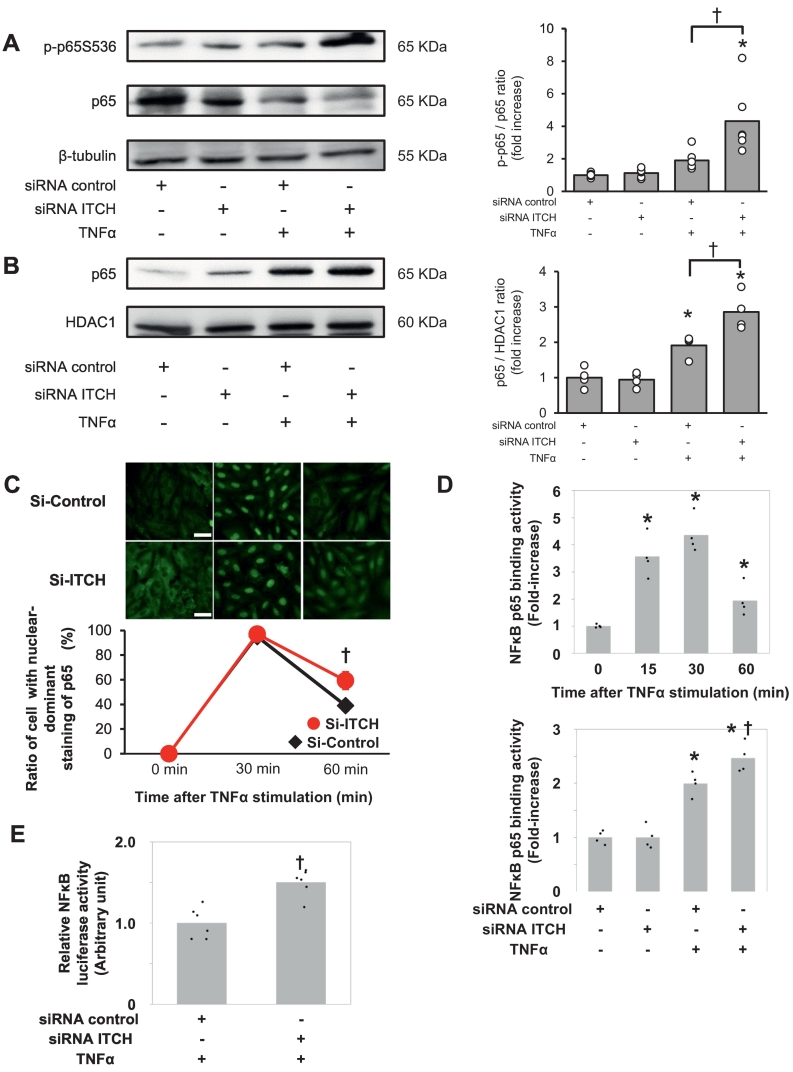
Knockdown of ITCH affects the subcellular localization of NF-κB after TNFα stimulation. (A) Representative western blot analysis of phospho-p65 and p65 in siRNA-control- or siRNA-ITCH-transfected H9C2 cells 15 min after TNFα stimulation (20 ng/mL). Quantification of phospho-p65 protein levels in siRNA-control- or siRNA-ITCH-transfected H9C2 cells 15 min after TNFα stimulation (*n* = 6 per group). (B) Representative western blot analysis of p65 in nuclear fraction in siRNA-control- or siRNA-ITCH-transfected H9C2 cells 15 min after TNFα stimulation (20 ng/mL). Quantification of p65 protein levels in nuclear fraction was significantly higher in ITCH-knockdown H9C2 cells than in control-siRNA-transfected H9C2 cells 15 min after TNFα stimulation (*n* = 4 per group). (C) siRNA-ITCH transfected H9C2 cells accelerate the nuclear translocation of p65 60 min after TNFα (20 ng/mL) stimulation when compared with siRNA-control transfected H9C2 cells (scale bars; 50 μm, *n* = 6 per group). Fifty cells were counted for the subcellular localization of the p65 subunit in Fig. 4B image and classified into nuclear-dominant (nucleus > cytoplasm) or cytoplasmic-dominant (nucleus ≤ cytoplasm). (D) Quantification of DNA binding activity of NFκB p65 from nuclear extracts. Time course of DNA binding activity of NFκB p65 after TNFα stimulation (20 ng/mL) (*n* = 4 per group). Quantification of DNA binding activity of NFκB p65 was significantly higher in ITCH-knockdown H9C2 cells than in control-siRNA-transfected H9C2 cells 60 min after TNFα stimulation (n = 4 per group). (E) Quantification of NFκB activities using luciferase assay. Quantification of NFκB activities was significantly higher in ITCH-knockdown H9C2 cells than in control-siRNA-transfected H9C2 cells 30 min after TNFα stimulation (*n* = 6 per group). Data are expressed as mean ± standard error of the mean (SEM) (*P < 0.05 *vs.* siRNA-control-transfected H9C2 cells, †P < 0.05 *vs.* siRNA-control-transfected H9C2 cells after TNFα stimulation by Student's *t*-test or Tukey-Kramer analysis). TNFα, tumor necrosis factor alpha.

Next, we stimulated H9C2 cells with TNFα following ITCH knockdown. We observed that the phosphorylation level of p65 was significantly higher in H9C2 cells transfected with ITCH-siRNA than in H9C2 cells transfected with control-siRNA after TNFα stimulation (Fig. 4A). In addition, the protein expression levels of p65 in nuclear fraction was significantly higher in ITCH-Knockdown H9C2 cells than in control-siRNA-transfected H9C2 cells after TNFα stimulation (Fig. 4B). It has been previously reported that the nuclear translocation of p65 is an important step in transcriptional activity following TNFα stimulation [24]. In addition, LPS-mediated activation of NF-κB is modulated by paracrine TNFα signaling between cells [25]. Therefore, we examined whether ITCH knockdown affected the subcellular localization of p65 after TNFα stimulation. Sixty minutes after TNFα stimulation, siRNA-ITCH-transfected H9C2 cells showed accelerated nuclear translocation of p65 when compared with control siRNA-transfected H9C2 cells (Fig. 4C). Next, we examined the effect of ITCH-Knockdown on DNA binding activity of NFκB p65 using Trans AM NFκB p65 assay kit. NFκB p65 binding activity reached the peak at 30 min and decreased at 60 min after TNFα stimulation. NFκB p65 binding activity 60 min after TNFα stimulation was significantly higher in ITCH-Knockdown H9C2 cells than in control siRNA-transfected H9C2 cells (Fig. 4D). Relative NFκB luciferase activity 30 min after TNFα stimulation was also significantly higher in ITCH-Knockdown H9C2 cells than in control siRNA-transfected H9C2 cells (Fig. 4E). These results suggested that ITCH modulates the activation of the NF-κB signaling pathway in cardiomyocytes.

### 3.2 *In vivo* study

#### 3.2.1 Interactions between ITCH and NF-κB signaling regulators in mouse heart

**Fig. 5:**
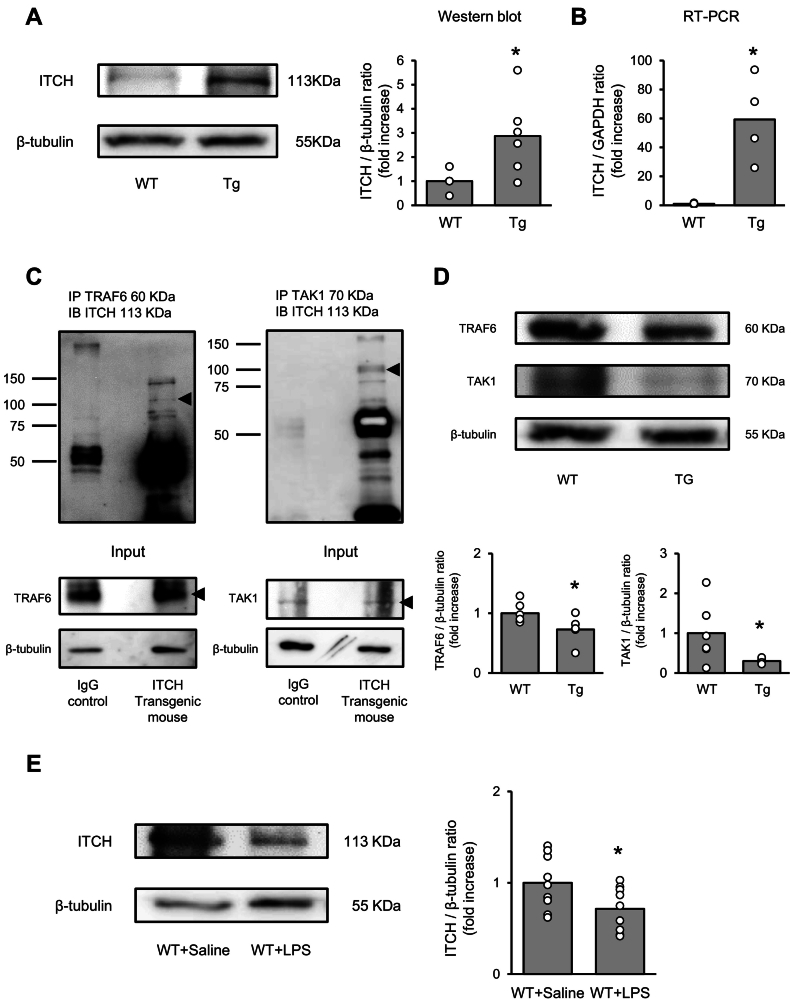
ITCH interacts with key regulators of NF-κB signaling *in vivo*. (A) Protein expression levels of ITCH in the heart ventricles of WT and ITCH-Tg mice examined by western blot analysis (n = 6 per group). (B) Comparison of mRNA expression of *ITCH* between WT and ITCH-Tg mice (n = 4 per group). (C) Immunoprecipitation shows that ITCH interacts with TRAF6 and TAK1. Western blots with ITCH antibody after immunoprecipitation showing interactions of ITCH with these proteins in mouse myocardium lysates. (D) Expression levels of TRAF6 and TAK1 in the heart of WT and ITCH-Tg mice in western blot analysis (*n* = 6 per group). (E) Protein expression levels of ITCH in the heart after LPS (50 mg/kg) intraperitoneal injection (*n* = 9 per group). Results are presented as the mean ± standard error of the mean (SEM) (*P < 0.05 *vs.* WT mice by Student's t-test or Tukey-Kramer analysis). LPS, lipopolysaccharide; TAK1, transforming growth factor-β activated kinase 1; TRAF6, tumor necrosis factor receptor-associated factor 6; WT, wild-type.

To clarify the role of ITCH in septic cardiomyopathy, WT and ITCH-Tg mice were intraperitoneally injected with LPS or saline, respectively. Protein and mRNA expression levels of ITCH were higher in ITCH-Tg mice than in WT mice (Fig. 5A and B). Immunoprecipitation was performed to clarify the interaction between ITCH and TAK1/TRAF6 in the myocardium. As shown in Fig. 5C, ITCH interacted with TRAF6 and TAK1 in the myocardium. To determine whether cardiac overexpression of ITCH modulated the protein expression levels of TRAF6 and TAK1, western blot analysis was performed. As shown in Fig. 5D, the protein levels of TRAF6 and TAK1 were lower in ITCH-Tg mice than in WT mice. Next, we examined changes in the endogenous expression levels of ITCH following LPS injection (50 mg/kg) and found that protein expression levels of ITCH were reduced (Fig. 5E).

#### 3.2.2 Improved cardiac function and survival rate in cardiac-specific ITCH overexpression mice following LPS intraperitoneal injection

**Fig. 6:**
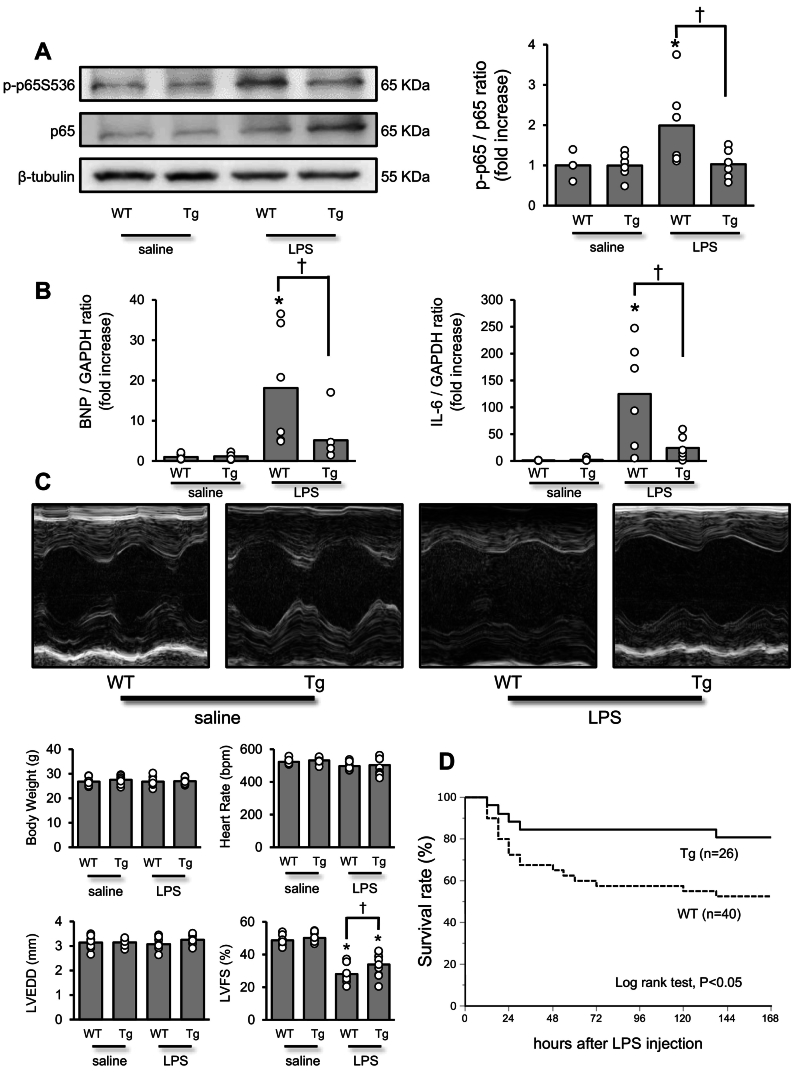
Cardiac-specific ITCH overexpression preserves cardiac contraction and improves survival rate after intraperitoneal LPS administration. (A) Representative western blot image of phospho-p65 and p65 in WT and ITCH-Tg mice after intraperitoneal saline or LPS (50 mg/kg) injection 6 h after administration. Quantification of phospho-p65 protein levels in WT and ITCH-Tg mice after intraperitoneal saline or LPS injection (n = 6 per group). (B) Quantitative analyses of *Bnp* and *IL-6* gene expression in WT and ITCH-Tg mice after intraperitoneal saline or LPS (50 mg/kg) injection 6 h after administration (n = 6 per group). (C) Representative images and analysis of cardiac function using echocardiography in WT and ITCH-Tg mice after intraperitoneal saline or LPS injection (*n* = 10 per group). (D) Survival curves using Kaplan-Meier analysis in WT and ITCH-Tg mice after intraperitoneal LPS injection. Data are expressed as mean ± standard error of the mean (SEM) (*P < 0.05 *vs.* WT mice after saline intraperitoneal injection; †P < 0.05 *vs.* WT mice after LPS intraperitoneal injection by Student's *t*-test or Tukey-Kramer analysis). LPS, lipopolysaccharide; WT, wild-type.

We examined the phosphorylation levels of p65 in WT and ITCH-Tg mice after an intraperitoneal injection of LPS (50 mg/kg). As shown in Fig. 6A, the phosphorylation level of p65 was lower in ITCH-Tg mice than in WT mice after intraperitoneal LPS injection. The mRNA expression levels of *Bnp* and *IL-6* were significantly suppressed in ITCH-Tg mice compared to those in WT mice after LPS intraperitoneal injection (Fig. 6A). Representative M-mode echocardiograms obtained 6 h after intraperitoneal injection of LPS (50 mg/kg) are shown in Fig. 6C. LV fractioning shortening (LVFS) of WT mice was significantly decreased after LPS intraperitoneal injection but was ameliorated in ITCH-Tg mice. Finally, we compared the survival rate of ITCH-Tg and WT mice after intraperitoneal injection of LPS (25 mg/kg). The survival rate after LPS intraperitoneal injection was significantly higher in ITCH-Tg mice than in WT mice (Fig. 6D).

These results suggest that ITCH in the myocardium plays a protective role in the development of septic cardiomyopathy by regulating the NF-κB signaling pathway after intraperitoneal injection of LPS.

## 4 Discussion

### 4.1 Main findings

**Fig. 7:**
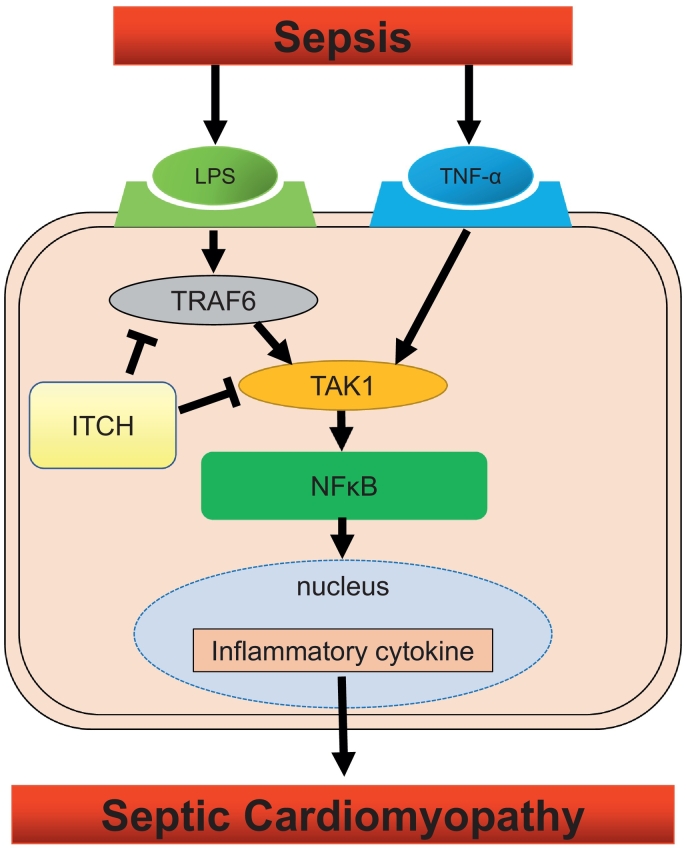
A schema representing ITCH-mediated inhibition of septic cardiomyopathy development.

The main findings of the present study are as follows: (1) protein expression of ITCH was decreased in response to LPS or TNFα in cardiomyocytes; (2) ITCH interacted with TRAF6 and TAK1, regulating their protein expression *in vitro* and *in vivo*; (3) ITCH also bound to A20 and CYLD in H9C2 cardiomyocytes; (4) ITCH modulated phosphorylation of p65, nuclear translocation of p65, and subsequent *IL-6* expression in cardiomyocytes; (5) ITCH-Tg mice exhibited lower protein expression levels of TRAF6 and TAK1 than WT mice; (6) cardiac-specific overexpression of ITCH preserved left ventricular systolic function and improved survival rate following LPS intraperitoneal injection. Fig. 7 represents a schematic representation of the current study. Our results suggest that ITCH plays a protective role in the development of septic cardiomyopathy in cardiomyocytes.

### 4.2 Downregulation of ITCH in response to LPS and TNFα

The HECT-type E3 ligase ITCH was first identified in a mutant mouse with an aberrant immunological phenotype [26] and was reported to play a critical role in the cessation of inflammation by suppressing the NF-κB pathway in immune cells [11], [23]. Thus, ITCH protein expression might have an important role in inflammation. The E3 ligase activity of ITCH is reportedly modulated by its expression level and phosphorylation by interacting with adaptor proteins, such as c-Jun N-terminal kinase, which relieves the autoinhibitory conformation of the WW-linker to the HECT domain of ITCH [27]. We have previously reported that ITCH was downregulated in response to oxidative stress, such as doxorubicin and hydrogen peroxide stimulation, in cardiomyocytes through auto-ubiquitylation [12]. However, the effect of LPS and TNFα stimulation on the protein expression level of ITCH in cardiomyocytes has not been established. In the present study, we revealed that the protein expression level of ITCH was serially decreased following LPS and TNFα stimulation in H9C2 cells. Furthermore, the protein expression level of ITCH decreased following intraperitoneal injection of LPS *in vivo*. These results suggest that ITCH is downregulated in septic cardiomyopathy.

We also examined serial changes in the protein expression levels of A20 and CYLD, which are ITCH binding partners. Interestingly, the trends in these protein expression levels differed depending on the stimulation, *i.e.*, LPS and TNFα. A20 was reported to inhibit coxsackievirus B3-induced myocarditis *via* the NF-κB pathway [28], suggesting that upregulation of A20 relieves LPS-induced inflammation. Despite A20 upregulation, LPS induces inflammation in cardiomyocytes. It can be postulated that A20 upregulation failed to inhibit LPS-induced inflammation in cardiomyocytes owing to the lack of ICTH, an essential component of the A20 ubiquitin-editing complex [15]. In addition, CYLD plays a causal role in inhibiting the NF-κB signaling pathway, and a previous report has shown that CYLD knockdown could increase LPS-stimulated NF-κB activation [29]. Therefore, insufficient CYLD levels following TNFα stimulation might also contribute to the development of septic cardiomyopathy. Notably, both LPS- and TNFα-stimulated cardiomyocytes exhibited ITCH downregulation. These findings support our hypothesis that the development of septic cardiomyopathy is associated with insufficient ITCH-dependent inhibition of the NF-κB pathway.

### 4.3 Interaction between ITCH and key regulators of NF-κB signaling in cardiomyocytes

Based on the ligand, numerous mechanisms can activate the NF-κB pathway [30]. After LPS binds to Toll-like receptor 4, TRAF6 is activated to transduce signals [31]. TRAF6, a RING-type E3 ligase, complexes with TAK1 and catalyzes the K-63 polyubiquitin chain, which can serve as a scaffold to link signaling protein complexes [31]. Activated TAK1 leads to NF-κB activation by phosphorylating IκB kinase (IKK) β [32]. Therefore, TRAF6 and TAK1 are key components of the LPS-induced NF-κB signaling pathway.

ITCH can reportedly promote TRAF6 deubiquitination in osteoclasts [14]. Previous reports have demonstrated that A20 inhibits TRAF6 signaling by inhibiting TRAF6 interaction with E2, removal of TRAF6 K63-linked ubiquitin chains, and K48-linked degradative ubiquitylation [31]. In addition, CYLD has been reported to inhibit RANKL signaling by deubiquitylation of TRAF6 in preosteoclasts [33]. In the present study, ITCH interacted with A20 and CYLD in cardiomyocytes, and ITCH overexpression promoted TRAF6 deubiquitylation in cardiomyocytes. These results suggest that ITCH modulates TRAF6 deubiquitylation by interacting with A20 and CYLD in cardiomyocytes. In addition, the impact of ITCH on the protein expression of TRAF6 has not been previously reported. Herein, we showed that ITCH inhibited the protein expression level of TRAF6 in cardiomyocytes. Several reports have indicated that septic cardiomyopathy was ameliorated by inhibiting TRAF6 protein expression and subsequent NF-κB activation, suggesting the importance of TRAF6 protein expression [34], [35], [36]. Therefore, reduced TRAF6 expression may contribute to the inhibition of LPS-induced NF-κB activation in ITCH-Tg mice.

TAK1, a member of the family of mitogen-activated protein kinases, phosphorylates numerous downstream targets, including IKKβ, in NF-κB signaling [37]. The TAK1 complex, comprising TAK1 binding proteins 1, 2, and 3, is attached to K-63 linked ubiquitin chains by E3 ligases and activated after cellular stimulation [37], suggesting the importance of ubiquitylation in regulating TAK1 activation. Previous reports have demonstrated that ITCH sequentially catalyzes K48-linked ubiquitin chains on TAK1 and degrades it *via* the proteasome pathway after the removal of K63-linked ubiquitylation by CYLD [13]. In the present study, we demonstrated that ITCH overexpression could inhibit TAK1 expression in the heart. Therefore, ITCH may inhibit the activation of NF-κB signaling by decreasing the protein expression level of TAK1.

To enforce the inhibitory effect of ITCH on NF-κB signaling, we stimulated H9C2 cells with TNFα. Consistent with LPS stimulation, overexpression and knockdown studies demonstrated that ITCH inhibited TNFα-induced NF-κB signaling in H9C2 cells.

### 4.4 Association between ITCH and septic cardiomyopathy

Prolonged NF-κB activation in the heart reportedly triggers chronic inflammation and promotes heart failure through enhanced cytokine secretion [38]. Similarly, NF-κB activation plays a central role in the development of septic cardiomyopathy through direct damage to cardiomyocytes, calcium imbalance, mitochondrial dysfunction, and β-receptor downregulation [39]. Interleukin-6, a major NF-κB target gene, is produced by several cells, such as vascular endothelium, smooth muscle cells, cardiomyocytes, fibroblasts, and interstitial macrophages in the heart [40]. Cardiac-specific overexpression of IL-6 has been reported to deteriorate systolic dysfunction, inflammation, and apoptosis in mice after burn plus sepsis, whereas these changes were reportedly attenuated in IL-6 knockout mice [41]. Cardiac dysfunction after trauma-hemorrhage shock has been attributed to a local increase in IL-6 levels in cardiomyocytes [42]. These findings indicate that IL-6 production in cardiomyocytes contributes to cardiac dysfunction. TRAF6 deubiquitylation can be associated with the suppression of IL-6 production and inflammatory autoimmune disease [43]. We showed that ITCH overexpression reduced TRAF6 protein expression and promoted TRAF6 deubiquitylation in cardiomyocytes. Furthermore, ITCH overexpression in cardiomyocytes suppressed subsequent *IL-6* expression *in vivo*. There were no significant differences in cardiac fibrosis and macrophage infiltration between WT and ITCH-Tg mice after LPS administration (Fig. S2). Therefore, it can be speculated that ITCH preserved cardiac function by inhibiting IL-6 production in the myocardium.

To the best of our knowledge, we first report that cardiac-specific ITCH transgenic mice exhibit improved survival rates in septic cardiomyopathy. In the present study, we showed that cardiac-specific overexpression of ITCH preserved LVFS and improved survival rate after intraperitoneal LPS administration. These findings emphasize our hypothesis that ITCH plays a critical role in improving the survival rate and systolic function in septic cardiomyopathy.

This study has several limitations. First, we did not examine the chronic effect of ITCH on septic cardiomyopathy. Second, ubiquitination of TRAF6 and TAK1 was not completely assessed since it was complex process involved in K63-linked and K48-linked ubiquitination and de-ubiquitination. Further studies are required to reveal the precise mechanism by which ITCH inhibits septic cardiomyopathy.

## 5 Conclusions

ITCH modulated the NF-κB signaling pathway by interacting with key regulators of the NF-κB signaling pathway and improved the survival rate following intraperitoneal LPS administration. Our results suggest that ITCH may serve as a novel therapeutic target for preventing septic cardiomyopathy in patients with sepsis.

## Sources of funding

This study was supported by the Ministry of Education, Science, Sports, and Culture, Japan, and Grant-in-Aid for Scientific Research No. 21K16076 to Y. Otaki.

## Disclosures

None.

## Declaration of competing interest

The authors declare that they have no known competing financial interests or personal relationships that could have appeared to influence the work reported in this paper.

## Appendix A Supplementary data

Supplementary material Image 1
