# Supplementary material for: Cardiac-specific ITCH overexpression ameliorates septic cardiomyopathy via inhibition of the NF-κB signaling pathway
Source: J Mol Cell Cardiol Plus. 2022 Nov 2;2:100018. doi: 10.1016/j.jmccpl.2022.100018 (PMC11708253; doi:10.1016/j.jmccpl.2022.100018)

**ONLINE SUPPLEMENT**

**Cardiac-specific overexpression of ITCH attenuates septic cardiomyopathy through the inhibition of NFκB signaling pathway**

Yuji Saito, MD, PhD, Yoichiro Otaki, MD, PhD, Tetsu Watanabe, MD, PhD,

Shingo Tachibana, MD, Junya Sato, MD, Yuta Kobayashi, MD, Tomonori Aono, MD,

Jun Goto, MD, PhD, Masahiro Wanezaki, MD, PhD, Daisuke Kutsuzawa, MD, PhD,

Shigehiko Kato, MD, PhD, Harutoshi Tamura, MD, PhD, Satoshi Nishiyama, MD, PhD,

Takanori Arimoto, MD, PhD, Hiroki Takahashi, MD, PhD, and Masafumi Watanabe, MD, PhD

Department of Cardiology, Pulmonology, and Nephrology, Yamagata University School of Medicine, 2-2-2 Iida-Nishi, Yamagata, Japan 990-9585.

**Short title:** ITCH Ameliorates Septic-Cardiomyopathy

**Methods**

**Materials and Reagents**

As described previously [1], pRK5-ITCH (mouse) was a kind gift from Dr. H. Kamata (Hiroshima University, Japan). Empty vector pcDNA3.1-positive, Stealth select small interfering RNA (siRNA) for rat ITCH and Stealth RNAi negative control Medium GC Duplex #2 were purchased from Invitrogen Corp (Carlsbad, CA, USA). The cocktail of two duplexes was used for ITCH silencing, i.e. 1) sense 5’-UAGGCCGCUGAAUACUGUCAGUCAA-3’ and antisense 5'-UUGACUGACAGUAUUCAGCGGCCUA-3’and 2) sense 5'-CCCUGGAUGGGAGAAGAGAACUGAU-3’ and antisense 5'-AUCAGUUCUCUUCUCCCAUCCAGGG-3’.

Mouse monoclonal anti-TAK1 (1:1000 dilution, sc-7967), mouse monoclonal anti-A20 (1:1000 dilution, sc-166692) and mouse monoclonal anti-TRAF6 (1:1000 dilution, sc-8409) were purchased from Santa Cruz Biotechnology (Santa Cruz, CA, USA). Mouse monoclonal anti-ITCH (1:1000 dilution, #611198) was purchased from BD Biosciences (San Jose, CA, USA). Rabbit monoclonal anti-p65 (1:1000 dilution, #8242), rabbit monoclonal anti-phospho-p65 (Ser536, 1:1000 dilution, #3033), rabbit monoclonal anti-IκBα (1:1000 dilution, #4812), rabbit monoclonal anti-CYLD (1:1000 dilution, #8462), and rabbit polyclonal anti-β-tubulin (1:1000 dilution, #2146) were purchased from Cell Signaling Technology (Danvers, MA, USA). Rabbit polyclonal anti-CYLD antibody (11110-1-AP) and Rabbit polyclonal anti-HDAC1 antibody (10197-1-AP) were purchased from Proteintech (Rosemont, IL, USA).

LPS from Salmonella was purchased from Sigma-Aldrich Corp. TNFα (210-TA-005) was purchased from R＆D Systems, Inc.

NanoLuc® Reporter Vector with NF-κB Response Element (pNL3.2.NF-κB-RE[NlucP/NF-κB-RE/Hygro] Vector) was purchased from Promega (Madison, WI, USA).

**Western blot analysis**

Cardiomyocytes were lysed in cold lysis buffer, and the collected proteins were extracted, as reported previously [1,2]. Equal amounts of protein were subjected to 8% sodium dodecyl sulfate-polyacrylamide gel electrophoresis (SDS-PAGE), and transferred to polyvinylidene difluoride membranes. Membranes were blocked with 20 mM Tris-HCl, pH 7.4, containing 150 mM NaCl, 0.1% Tween (TBS-T) and 5% milk or 5% bovine serum albumin (BSA). These membranes were probed with primary antibodies diluted in TBS-T. After treatment with secondary antibodies diluted in TBS-T containing 5% milk or 5% BSA, immunoreactive bands were detected using Luminata Forte Western HRP Substrate (Merck, Darmstadt, Hesse, Germany). Expression levels of protein were normalized by β-tubulin. The bands were analyzed by using Light Capture Ⅱ and CS Analyzer ver 3.0 software (ATTO, Tokyo, Japan).

To show subcellular localization of p65, we used NE-PER^®^ Nuclear and Cytoplasmic Extraction Kit (#78833, Thermo Fisher Scientific, Waltham, MA, USA). The component of nuclear fraction was normalized by HDAC1.

**Total RNA extraction**

Total RNA was extracted using TRIzol as described previously [1]. First-strand cDNA was synthesized from 1 μg RNA using oligo (dT) primers and Superscript IV reverse transcriptase (#18090, Thermo Fisher Scientific, Waltham, MA, USA).
**Real-time reverse transcription-polymerase chain reaction (RT-PCR)**

Real-time PCR was performed with SsoAdvanced Universal SYBR Green Supermix (BIO RAD, Hercules, CA, USA) in 20 μl using StepOnePlus (Thermo Fisher Scientific, Waltham, MA, USA) as described previously [1]. The following PCR primers were used: mouse brain natriuretic peptide (*Bnp*) 5'- GCCAGTCTCCAGAGCAATTC-3' (forward) and 5'-TCTTTTGTGAGGCCTTGGTC-3' (reverse)[1]; rat *Bnp* 5'- GAGAGAGCAGGACACCATCG-3' (forward) and 5'- CGGCGACAGATTAAGGAAAA-3' (reverse); mouse interleukin 6 (*Il-6*) 5'- CTCTGCAAGAGACTTCCATCCA-3' (forward) and 5'- GACAGGTCTGTTGGGAGTGG-3' (reverse)[3]; rat *Il-6* 5'- ACCACCCACAACAGACCAGT-3' (forward) and 5'- ACAGTGCATCATCGCTGTTC-3' (reverse); mouse *Gapdh* 5'- TATGATGACATCAAGAAGGT-3' (forward) and 5'-AAGAGTGGGAGTTGCTGTTG-3' (reverse)[1]; rat *Gapdh* 5'-TATGATGACATCAAGAAGGT-3' (forward) and 5'- AAGAATGGGAGTTGCTGTTG-3' (reverse). Mouse *Itch* primers (qMmuCID0010161) was purchased from BIO RAD (Hercules, CA, USA).　Expression levels of mRNA were normalized to glyceraldehyde-3-phosphate dehydrogenase.

**Immunoprecipitation**

Immunoprecipitation was performed as described previously [1]. Protein extracts were prepared in modified RIPA buffer. Cell extracts including 300 μg whole cell lysate from cardiomyocytes were precleared with protein A/G beads for 30 min. Precleared cell extracts were centrifuged by 4,500 g for 2 minutes, and supernatants were incubated with 2 μg antibody overnight at 4℃. Mouse monoclonal anti-TAK1, mouse monoclonal anti-A20, mouse monoclonal anti-TRAF6 and rabbit monoclonal anti-CYLD were used for immunoprecipitation. Normal rabbit or mouse IgG were used as a negative control. After incubation, supernatants were mixed with protein A/G beads and incubated for 2 hours at 4℃. Pellets were washed four times with RIPA buffer and resuspended in RIPA lysis buffer. Samples were subjected to 8% SDS-PAGE and immunoblotted by mouse anti-ITCH antibody (1:1000 dilution).

Selective isolation of ubiquitinated proteins was performed with Signal-Seeker^TM^ Ubiquitination Detection Kit (BK161-S, Cytoskeleton, Inc.). We performed western blot analysis to evaluate ubiquitinated proteins using anti-TRAF6 antibody.

**Immunofluorescence**

Immunofluorescence was performed as described previously [1]. H9C2 cells was fixed 4% paraformaldehyde in PBS for 10 minutes. Samples was blocked with PBS including 5% BSA and 1% Triton-X100 for 1 hour, incubated with primary antibodies overnight at 4°C. Rabbit monoclonal anti-p65 (1:1000 dilution, #8242) was used for primary antibodies. Samples were washed in PBS and incubated with secondary antibodies coupled to Alexa Fluor® 488 donkey anti-rabbit IgG (1:1000 dilution, Invitrogen, A24 21206). All images were obtained using a BX50 microscope (OLYMPUS, Tokyo, Japan). As described previously [4], we classified stained cells into nuclear-dominant (nucleus > cytoplasm) or cytoplasmic-dominant (nucleus ≤ cytoplasm) and evaluated subcellular localization of p65.

***LPS and TNFα Stimulation Protocol in H9C2 cell***

Rat H9C2 cardiomyocytes were cultured on collagen-coated dishes at 37°C in Dulbecco’s Modified Eagle Medium (DMEM) supplemented with 10% fetal bovine serum (FBS), penicillin (100 U/mL), and streptomycin (100 μg/mL) for 3-4 days. Cultured rat H9C2 cardiomyocytes that reached density 80-90% on the dish were treated with LPS (0.3 μg/μL) or TNFα (20 ng/mL) [5].

***Overexpression and knockdown of ITCH in H9C2 cell***

As reported previously [1], overexpression of ITCH by plasmid transfection and knockdown of ITCH by siRNA transfection were performed with Lipofectamine 3000 (Invitrogen, 29 Carlsbad, CA, USA) according to manufacturer’s protocol. Cardiomyocytes were incubated in Opti-MEM (Invitrogen, Carlsbad, CA, USA) for 2 hours before transfection. After incubation, cardiomyocytes were transfected with the plasmid or siRNA and incubated for 4-6 hours. After medium was changed, cardiomyocytes were kept in DMEM supplemented with 10% FBS, penicillin (100 U/mL), and streptomycin (100 mg/mL) for at least 24 hours. Later, experiments were performed.

***Cardiomyocyte isolation, culture, and treatments***

Cultured rat neonatal cardiomyocytes were prepared as described previously [1]. Hearts were excised from 1 to 2-day-old Sprague-Dawley rat pups, promptly after euthanasia by decapitation. To isolate cardiomyocytes, these tissues were incubated in a balanced salt solution containing collagenase type 2 (Worthington Biochemical, Lakewood, NJ, USA) for 10 minutes at 37°C. The digestion buffer was replaced 10 times, after which the tissues were completely digested. The unattached viable cells, which were enriched for neonatal rat cardiomyocytes, were cultured on collagen-coated dishes at 37°C in DMEM supplemented with 10% FBS, penicillin (100 U/mL), streptomycin (100 μg/mL), and 10 μM cytosine 1-β-D-arabinofuranoside (AraC), and cultured for 2 days. Then, cells were treated with TNFα (20 ng/mL).

***Experimental animal treatment***

As reported previously [1,2], experimental animals were handled according to the animal welfare regulations of Yamagata University, and the animal subjects committee of Yamagata University approved our study protocol (#R3035). Our study was conducted in accordance with the Guide for the Care and Use of Laboratory Animals published by the US National Institutes of Health.

***ITCH transgenic mouse generation***

As reported previously [1], transgenic mice with cardiac-specific overexpression of ITCH (ITCH-Tg mice) were created at Yamagata University using standard techniques.

***LPS-induced septic cardiomyopathy model in vivo***

Both wild-type (WT) and ITCH-Tg mice were randomly assigned to the control group (saline intraperitoneal injection) and sepsis group (50 mg/kg of LPS intraperitoneal injection) [6]. Both WT and ITCH-Tg mice were sacrificed at 6 hours after administration of LPS, and evaluated by western blot analysis and RT-PCR. LPS was administered at a dose of 25 mg/kg [7] in the observation of survival rate.

**Immunohistochemistry**

As described previously [8], paraffin-embedded heart sections were stained with CD11B antibody (21851-1-AP, Proteintech, Rosemont, IL, USA) with horseradish peroxidase (HRP)- conjugated IgG (PROMEGA, Madison, WI, the USA), followed by color development in 3,3'-diaminobenzidine tetrahydrochloride (Takara Bio Inc., Siga, Japan). Then nucleus was counterstained with hematoxylin.

**Histopathological examination**

As described previously [9], mice heart samples were fixed with 4% formalin and embedded in paraffin. Sections of 4-5 μm thickness were stained with Masson’s trichrome stain.

**NFκB binding activity assay**

p65 to DNA binding activity was measured using Trans AM p65 active motif assay kit (#40096, Active motif, CA, USA). Briefly, nuclear extracts were prepared using Nuclear Extracts Kit (#40010, Active motif, CA, USA). Samples were applied into NFκB 96 well stripe plate and treated according to manufacturer’s protocol. Absorbance at 450 nm was measured spectroscopically and referenced by absorbance at 650 nm.

**Luciferase reporter gene assay**

Transcriptional activation of NFκB was evaluated by luciferase reporter gene assay with pNL3.2.NF-κB-RE [NlucP/NF-κB-RE/Hygro] Vector, and the Nano-Glo® Luciferase Assay System (Promega, Madison, WI, USA). Expression vector transfection was performed using Lipofectamine 3000 Reagent (Invitrogen) according to the manufacturer’s instructions.

**Cardiac Function Analysis**

The cardiac function was evaluated by 2-dimensional echocardiography 6 hours after LPS or saline injection using Vevo2100 ultrasound echocardiography (Primetech Corporation, Tokyo, Japan). Heart Rate (HR), LV dimensions at end-diastole (LVEDD), and LV end-systole (LVESD) were measured digitally on the M-mode tracings. LV fractioning shortening (LVFS) was calculated as ([LVEDD - LVESD]/LVEDD)×100.

**Reference**

[1] J. Goto, Y. Otaki, T. Watanabe, Y. Kobayashi, T. Aono, K. Watanabe, M. Wanezaki, D. Kutsuzawa, S. Kato, H. Tamura, S. Nishiyama, T. Arimoto, H. Takahashi, T. Shishido, M. Watanabe, HECT (Homologous to the E6-AP Carboxyl Terminus)-Type Ubiquitin E3 Ligase ITCH Attenuates Cardiac Hypertrophy by Suppressing the Wnt/β-Catenin Signaling Pathway, Hypertension. 76 (2020) 1868–1878. https://doi.org/10.1161/HYPERTENSIONAHA.120.15487.

[2] Y. Otaki, H. Takahashi, T. Watanabe, A. Funayama, S. Netsu, Y. Honda, T. Narumi, S. Kadowaki, H. Hasegawa, S. Honda, T. Arimoto, T. Shishido, T. Miyamoto, H. Kamata, O. Nakajima, I. Kubota, HECT-type ubiquitin E3 ligase ITCH interacts with thioredoxin-interacting protein and ameliorates reactive oxygen species-induced cardiotoxicity, J. Am. Heart Assoc. 5 (2016) 1–18. https://doi.org/10.1161/JAHA.115.002485.

[3] G.G. Schiattarella, F. Altamirano, D. Tong, K.M. French, E. Villalobos, S.Y. Kim, X. Luo, N. Jiang, H.I. May, Z. V Wang, T.M. Hill, P.P.A. Mammen, J. Huang, D.I. Lee, V.S. Hahn, K. Sharma, D.A. Kass, S. Lavandero, T.G. Gillette, J.A. Hill, Nitrosative stress drives heart failure with preserved ejection fraction., Nature. 568 (2019) 351–356. https://doi.org/10.1038/s41586-019-1100-z.

[4] R. Tsuchiya, T. Tanaka, Y. Hozumi, T. Nakano, M. Okada, M.K. Topham, M. Iino, K. Goto, Downregulation of diacylglycerol kinase ζ enhances activation of cytokine-induced NF-κB signaling pathway, Biochim. Biophys. Acta - Mol. Cell Res. 1853 (2015) 361–369. https://doi.org/10.1016/j.bbamcr.2014.11.011.

[5] Y.L. Shen, Y.Z. Shi, G.G. Chen, L.L. Wang, M.Z. Zheng, H.F. Jin, Y.Y. Chen, TNF-α’ induces drp1-mediated mitochondrial fragmentation during inflammatory cardiomyocyte injury, Int. J. Mol. Med. 41 (2018) 2317–2327. https://doi.org/10.3892/ijmm.2018.3385.

[6] P.Y. Sips, T. Irie, L. Zou, S. Shinozaki, M. Sakai, N. Shimizu, R. Nguyen, J.S. Stamler, W. Chao, M. Kaneki, F. Ichinose, Reduction of cardiomyocyte S-nitrosylation by S-nitrosoglutathione reductase protects against sepsis-induced myocardial depression, Am. J. Physiol. - Hear. Circ. Physiol. 304 (2013) 1134–1146. https://doi.org/10.1152/ajpheart.00887.2012.

[7] L. Jia, Y. Wang, Y. Wang, Y. Ma, J. Shen, Z. Fu, Y. Wu, S. Su, Y. Zhang, Z. Cai, J. Wang, M. Xiang, Heme Oxygenase-1 in Macrophages Drives Septic Cardiac Dysfunction via Suppressing Lysosomal Degradation of Inducible Nitric Oxide Synthase, Circ. Res. 122 (2018) 1532–1544. https://doi.org/10.1161/CIRCRESAHA.118.312910.

[8] H. Machida, S. Inoue, A. Igarashi, S. Saitoh, K. Yamauchi, M. Nishiwaki, T. Nemoto, Y. Otaki, M. Sato, K. Sato, H. Nakano, S. Yang, K. Furuyama, H. Murano, Y. Ishibashi, T. Ota, T. Nakayama, Y. Shibata, M. Watanabe, Role of CC Chemokine Ligand 17 in Mouse Models of Chronic Obstructive Pulmonary Disease., Am. J. Respir. Cell Mol. Biol. 66 (2022) 428–438. https://doi.org/10.1165/rcmb.2021-0069OC.

[9] K. Watanabe, T. Narumi, T. Watanabe, Y. Otaki, T. Takahashi, T. Aono, J. Goto, T. Toshima, T. Sugai, M. Wanezaki, D. Kutsuzawa, S. Kato, H. Tamura, S. Nishiyama, H. Takahashi, T. Arimoto, T. Shishido, M. Watanabe, The association between microRNA-21 and hypertension-induced cardiac remodeling, PLoS One. 15 (2020) e0226053. https://doi.org/10.1371/journal.pone.0226053.

**Supplemental figure**

**
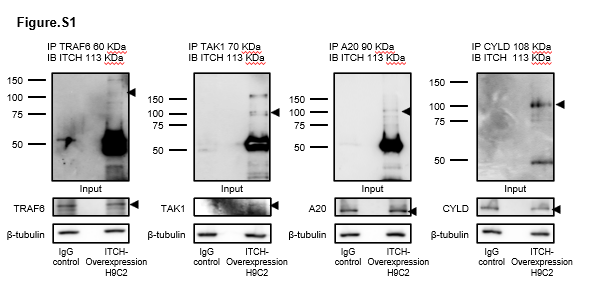
**

**Figure S1. Interactions between ITCH and other proteins such as TRAF6, TAK1, A20, and CYLD in ITCH-overexpressing H9C2 cells.**

Immunoprecipitation showing that ITCH interacts with TRAF6/TAK1/A20/CYLD in ITCH-overexpressing H9C2 cells. Western blots with ITCH antibody after immunoprecipitation shows interactions of ITCH with these proteins in ITCH-overexpressing H9C2 cell lysates. LPS, lipopolysaccharide; TAK1, transforming growth factor-β activated kinase 1; TRAF6, tumor necrosis factor receptor-associated factor 6.


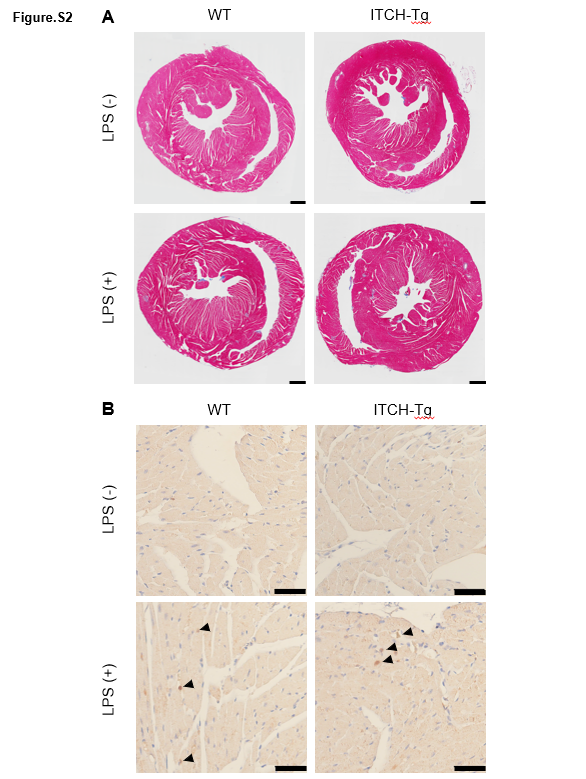


**Figure S2. Histopathological examination and immunohistochemistry after intraperitoneal LPS administration.**

(A) Representative images by Masson’s trichrome staining in mice heart samples after saline of LPS intraperitoneal administration. Scale bars = 500 μm. (B) Immunohistochemistry of heart sections after saline of LPS intraperitoneal administration using anti-CD11B (arrowheads). Scale bars = 50 μm. LPS, lipopolysaccharide; WT, wild-type.
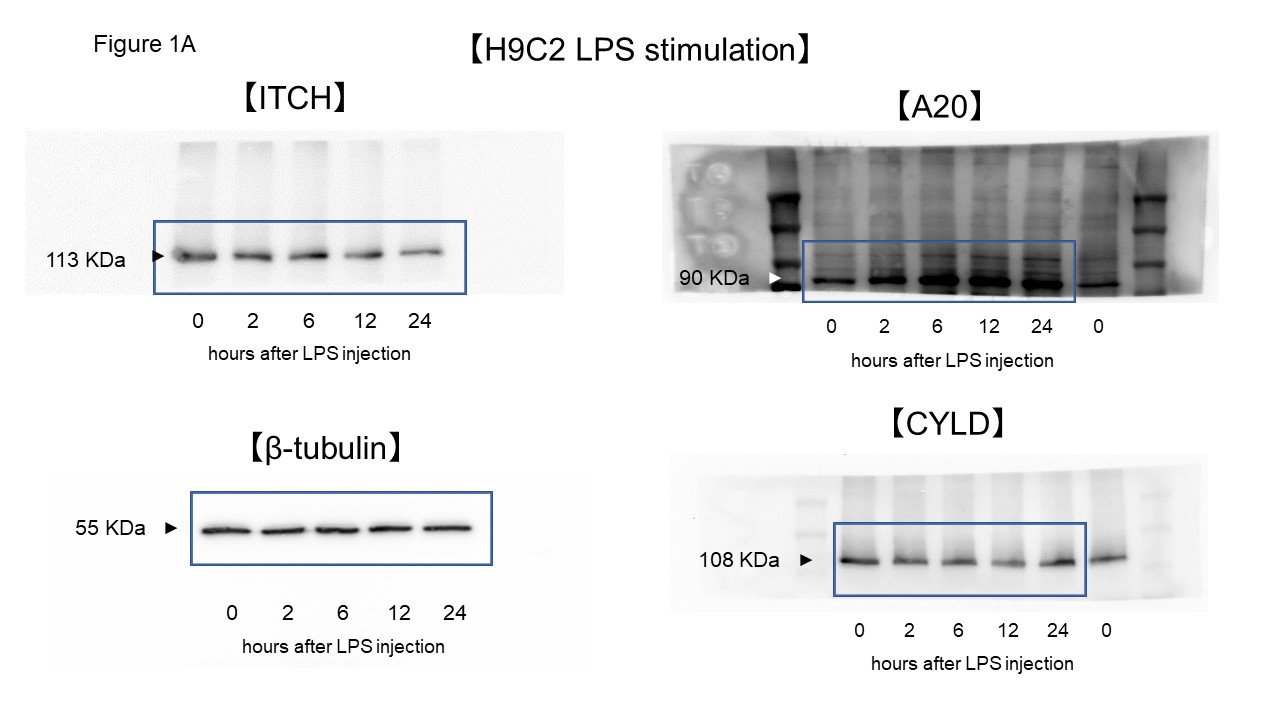


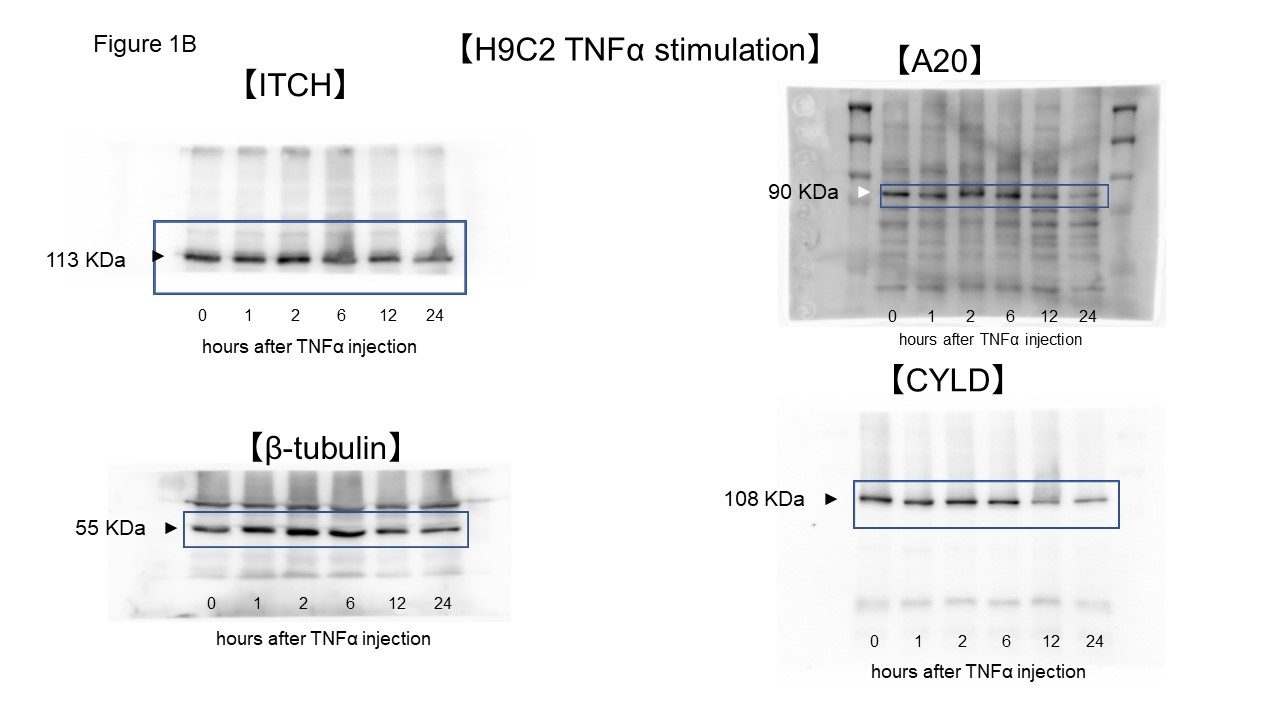


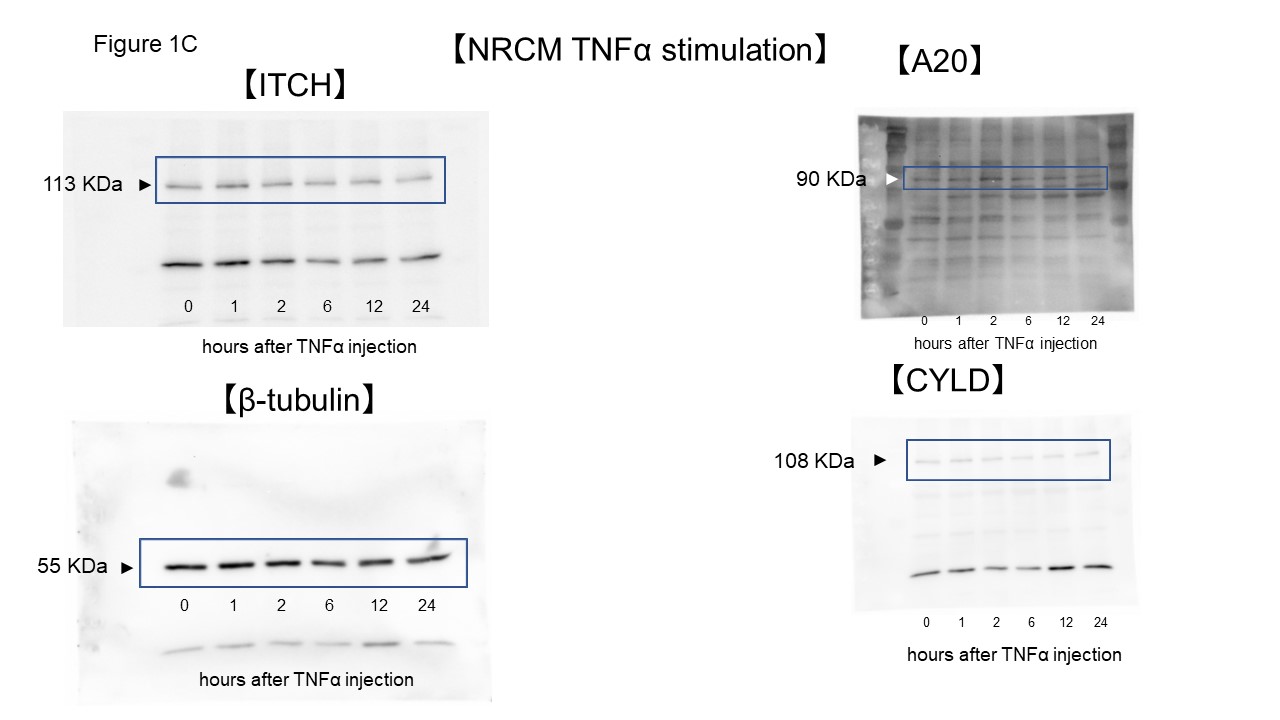


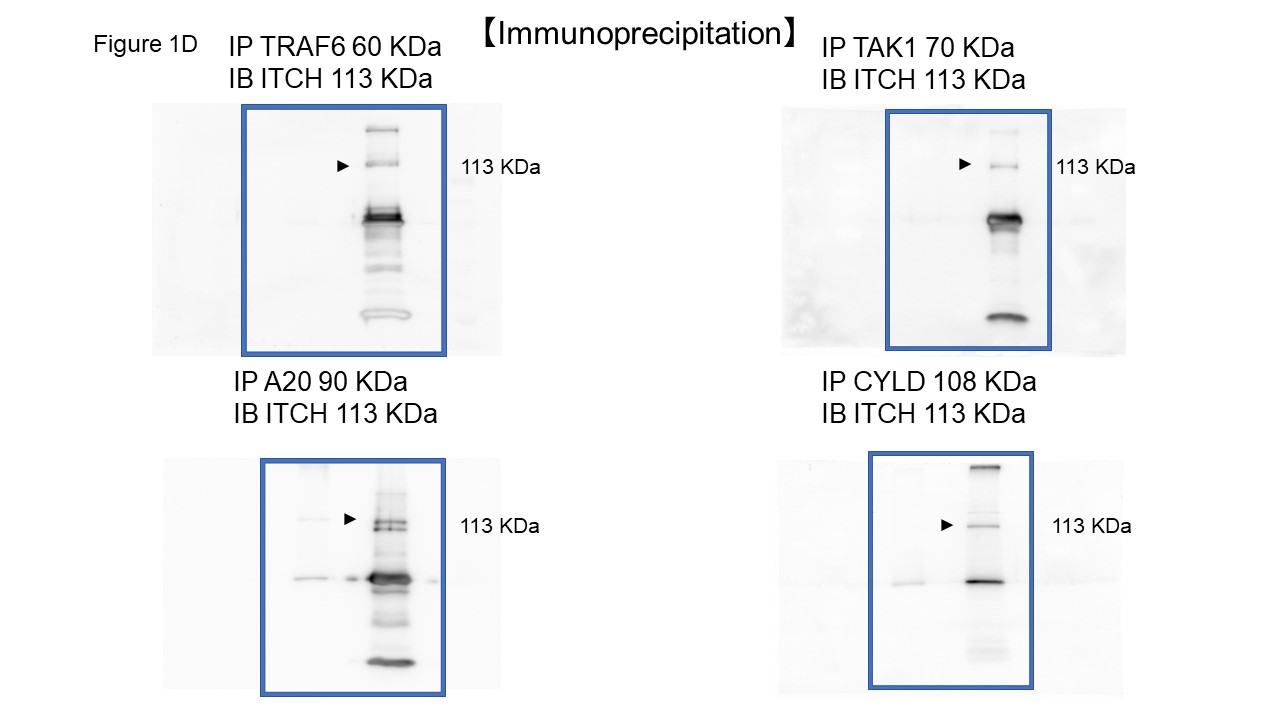


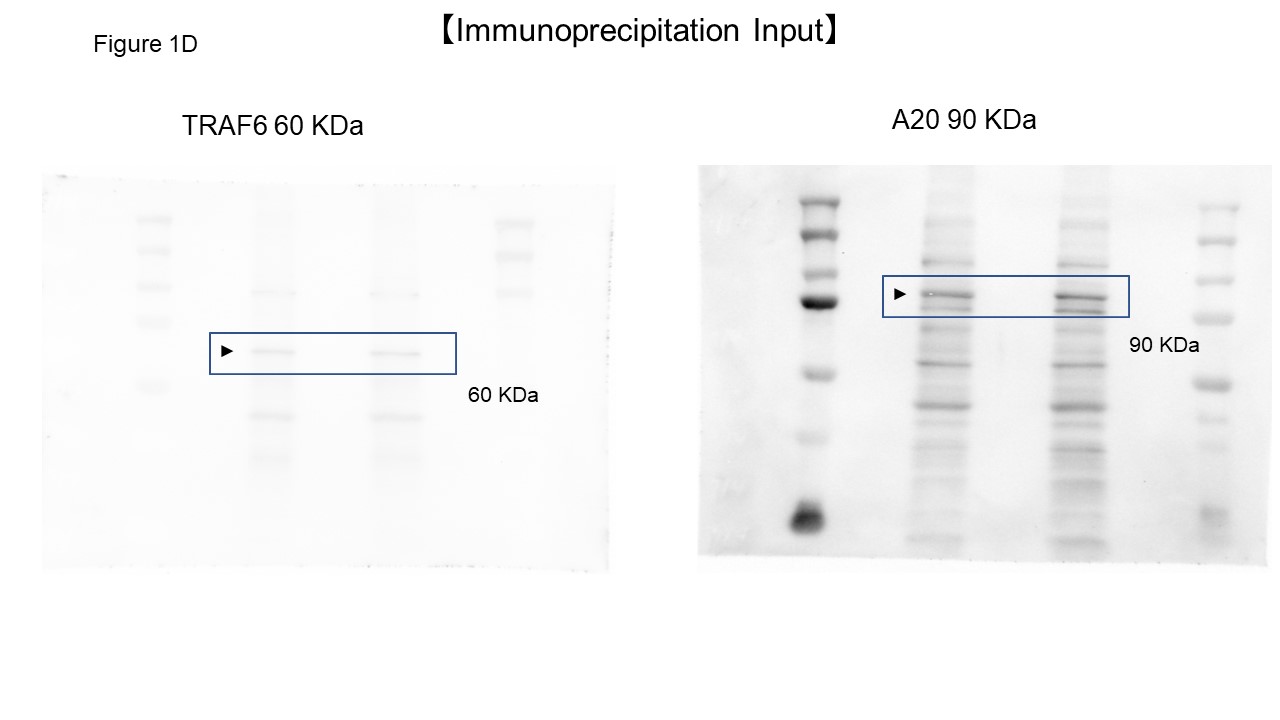


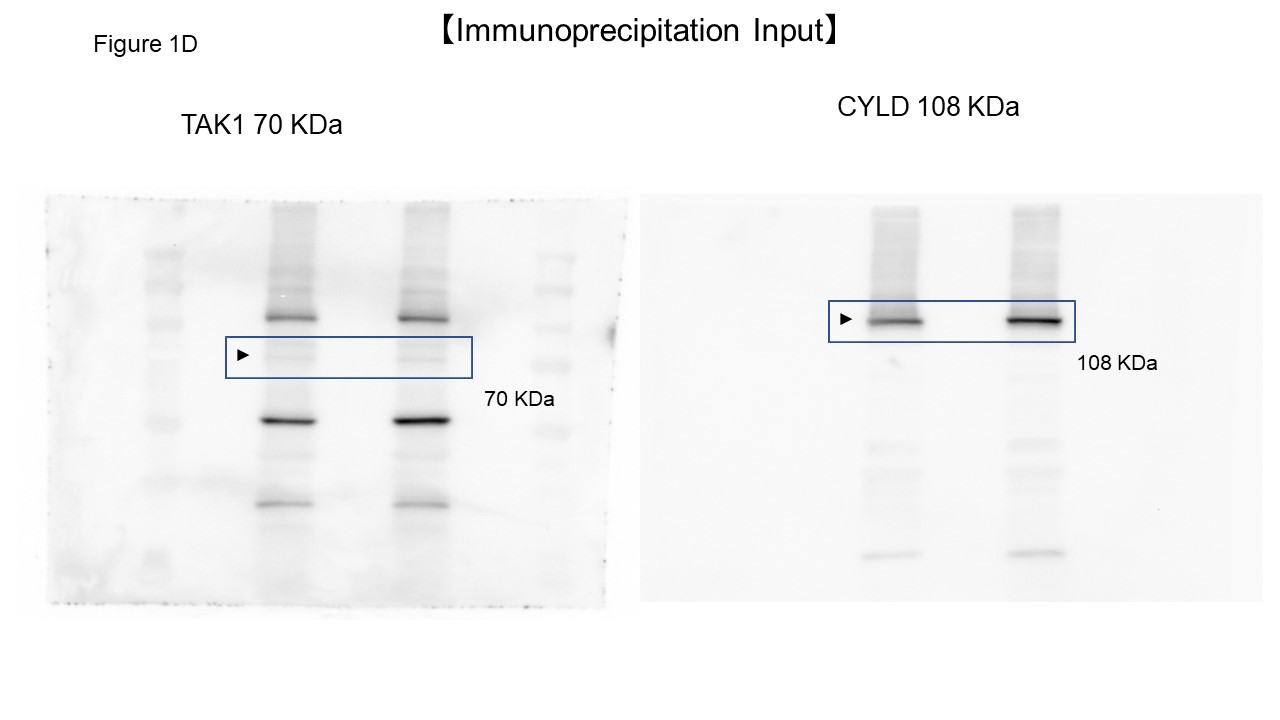


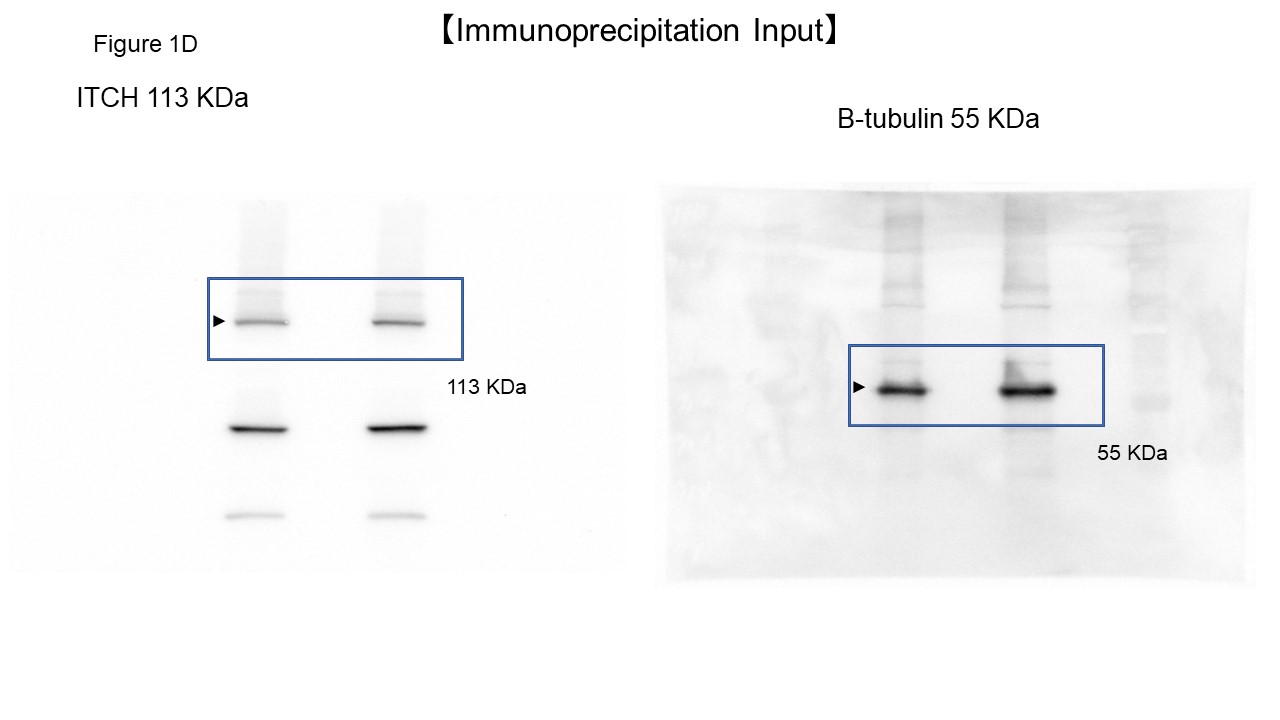


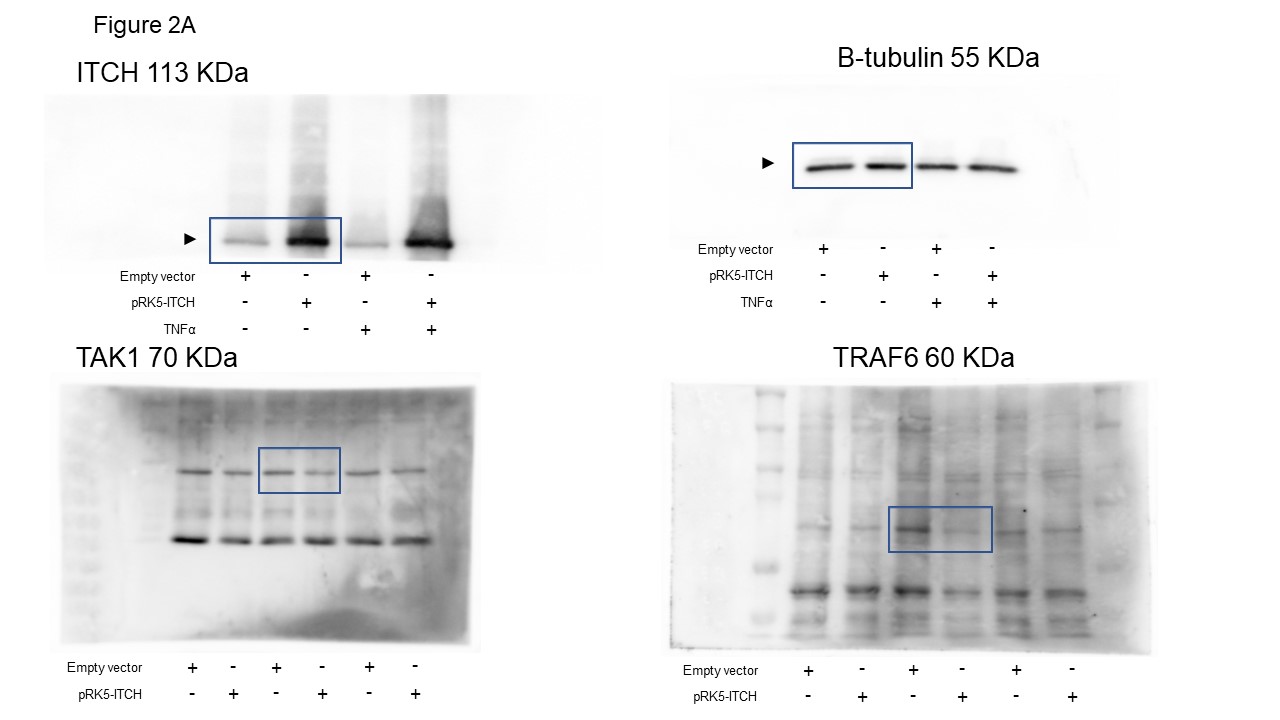


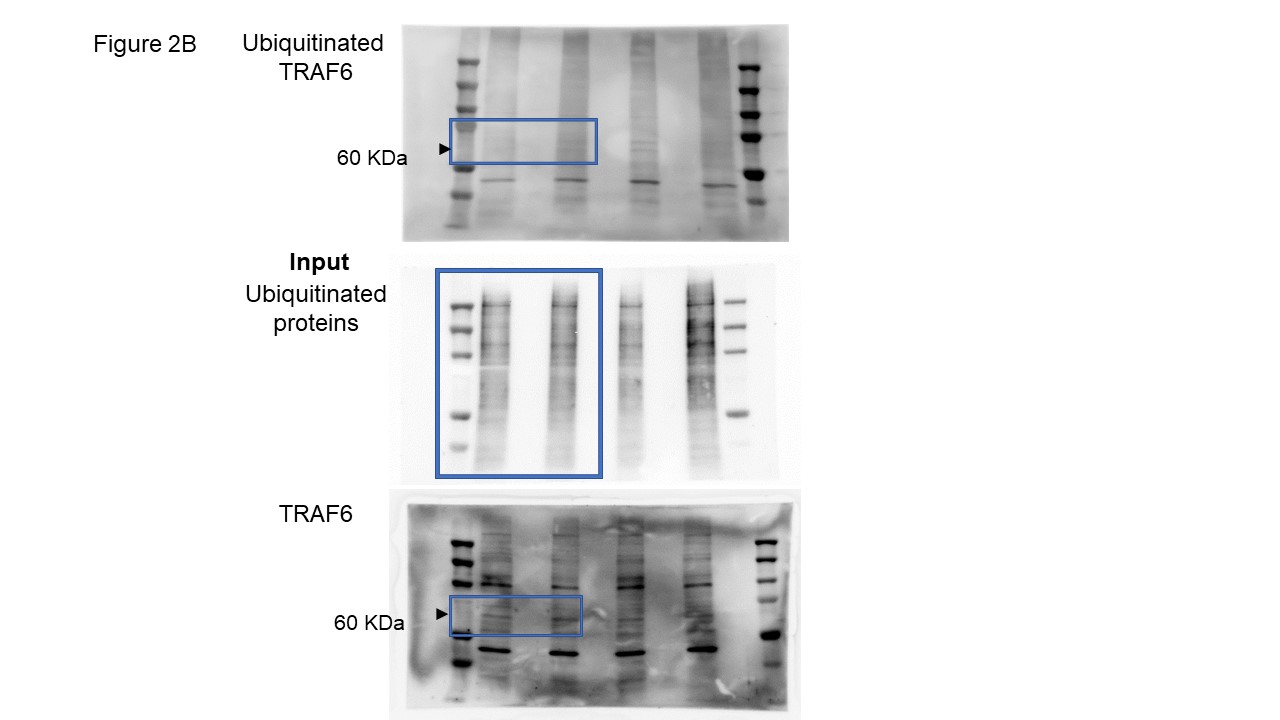


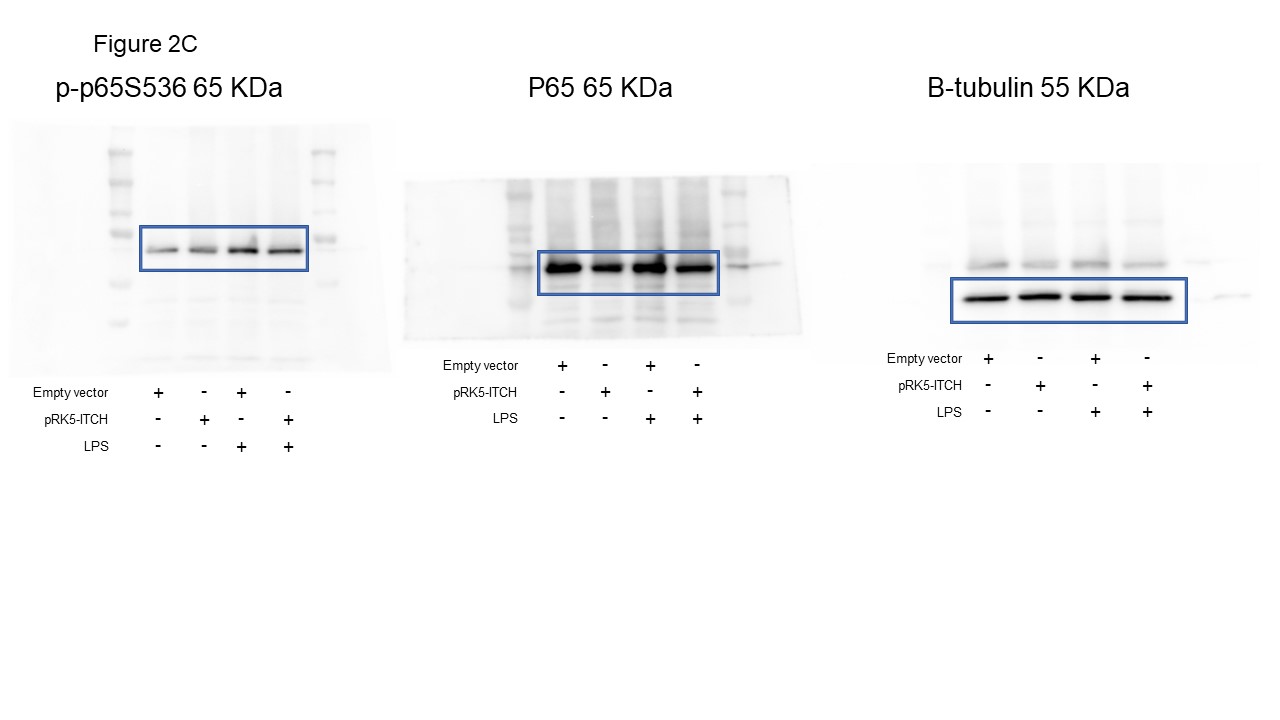


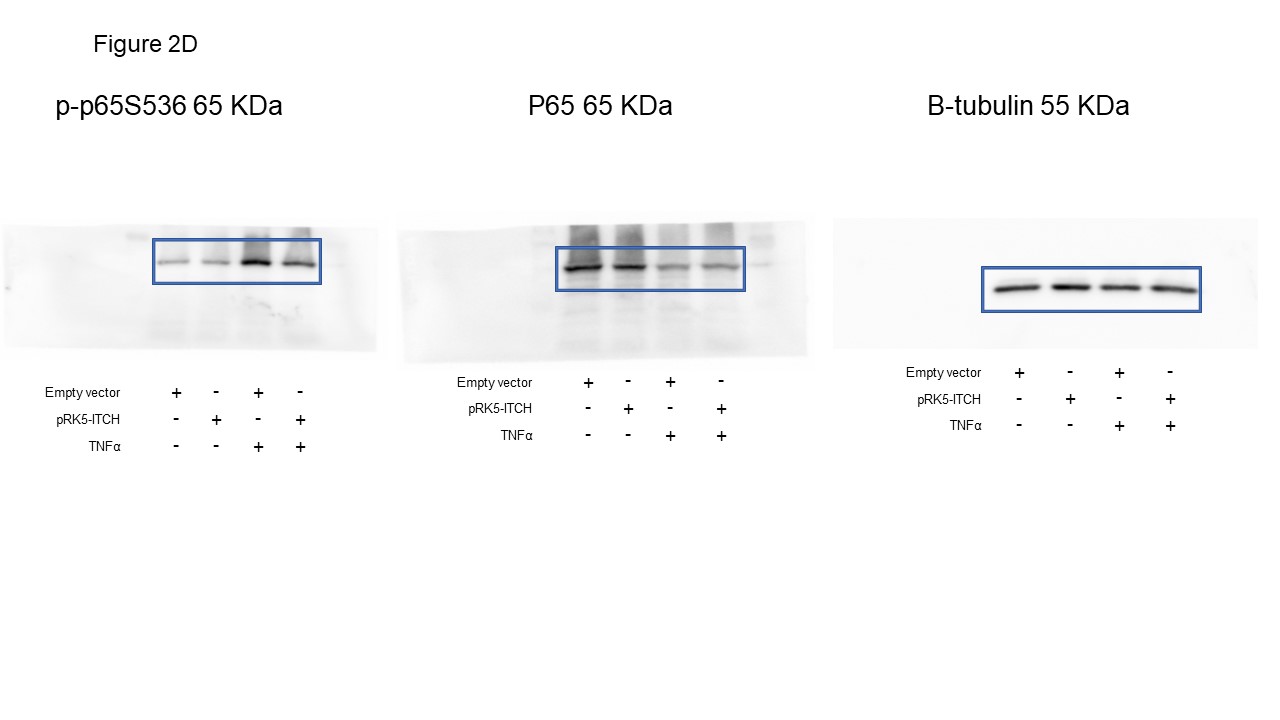


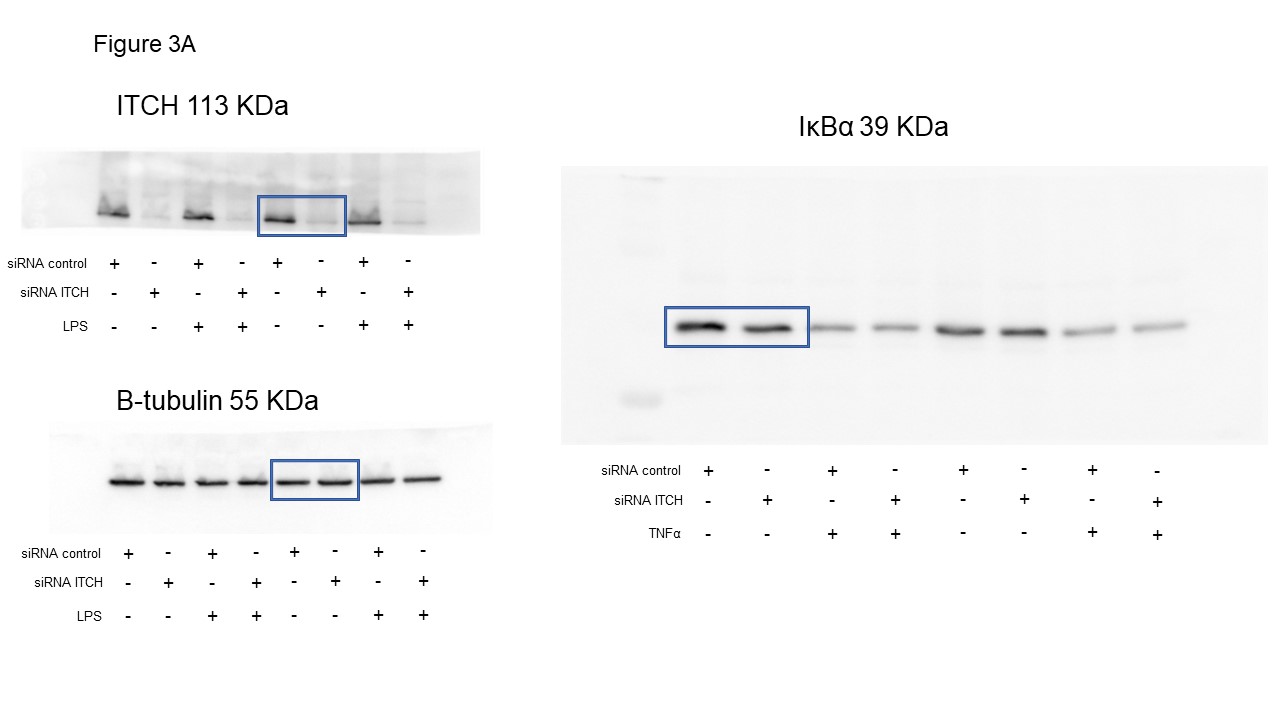


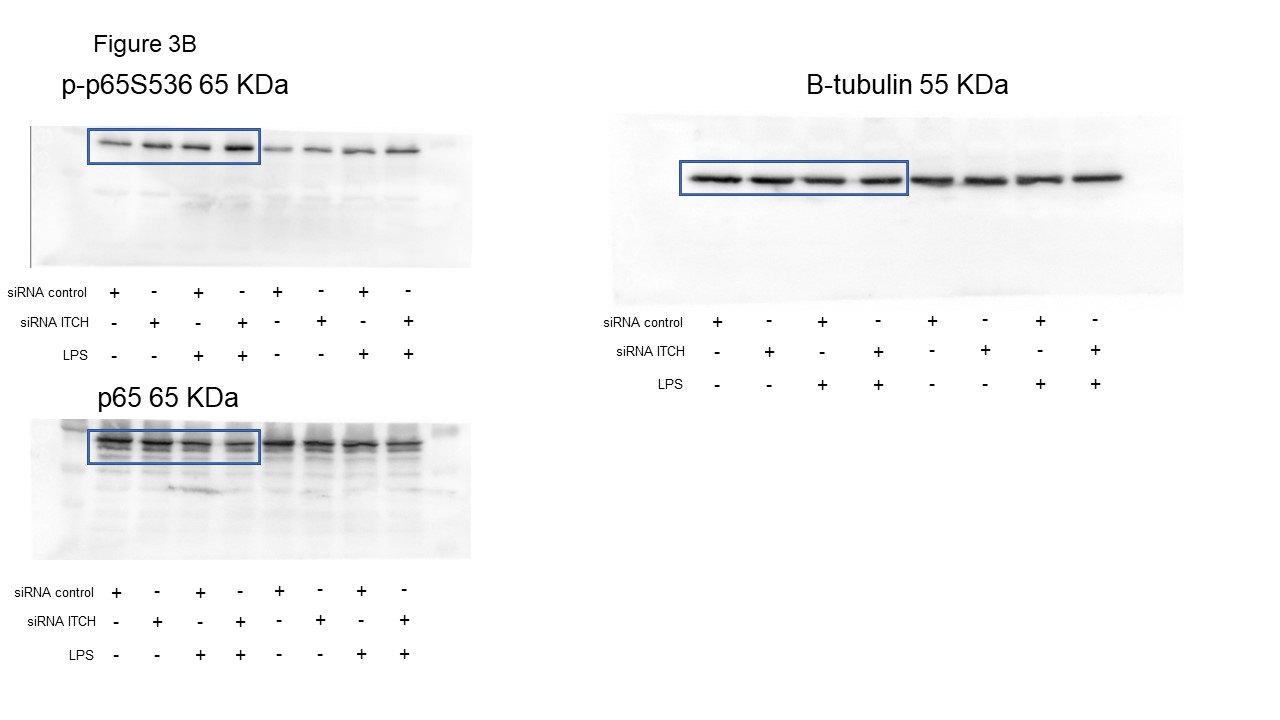


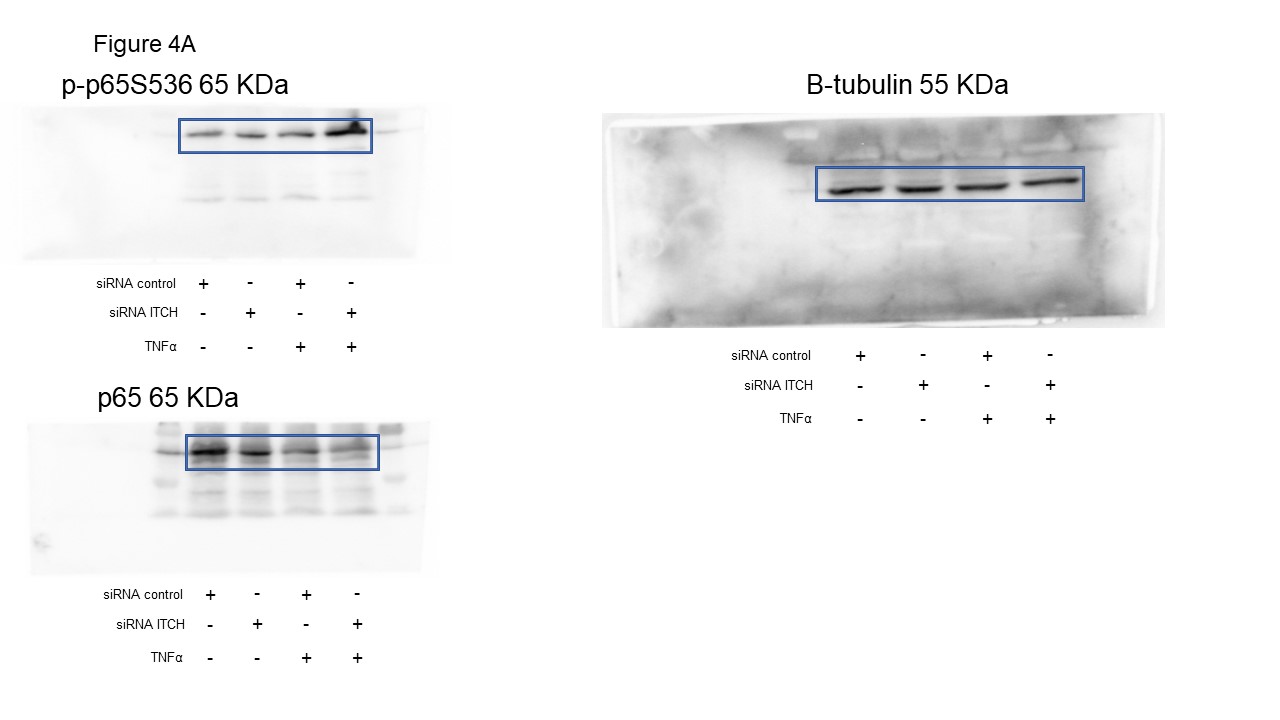


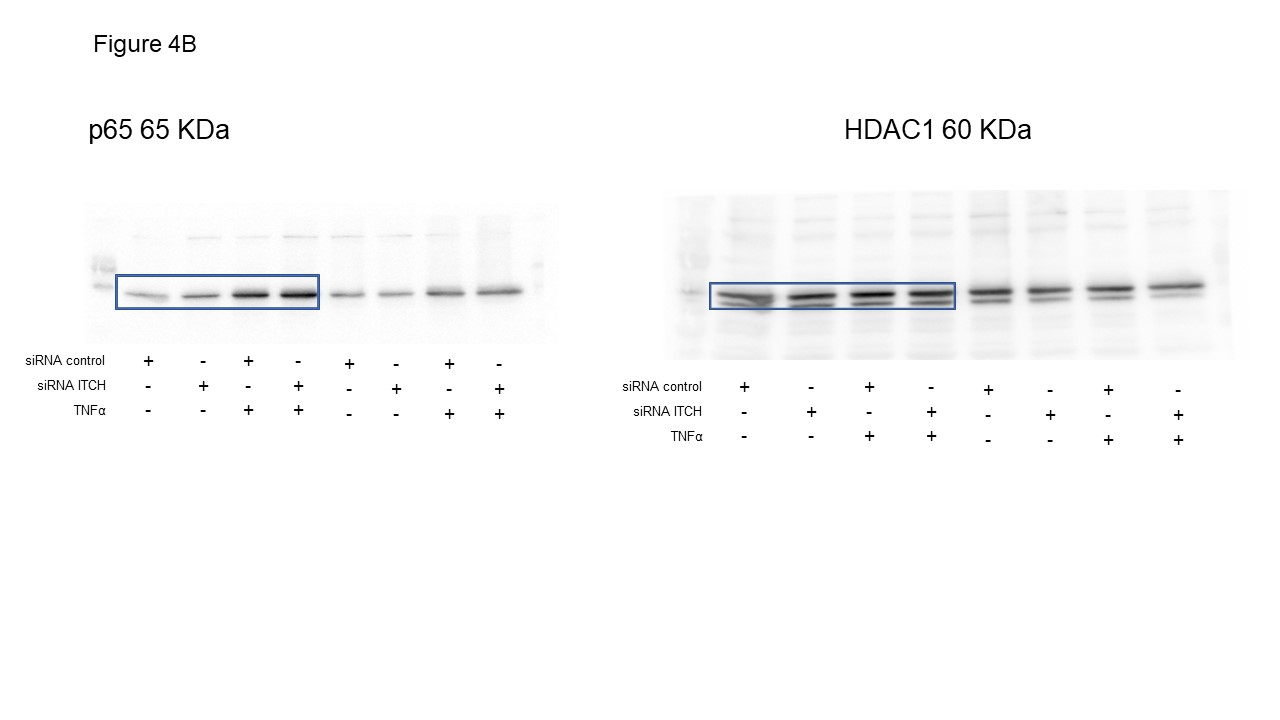


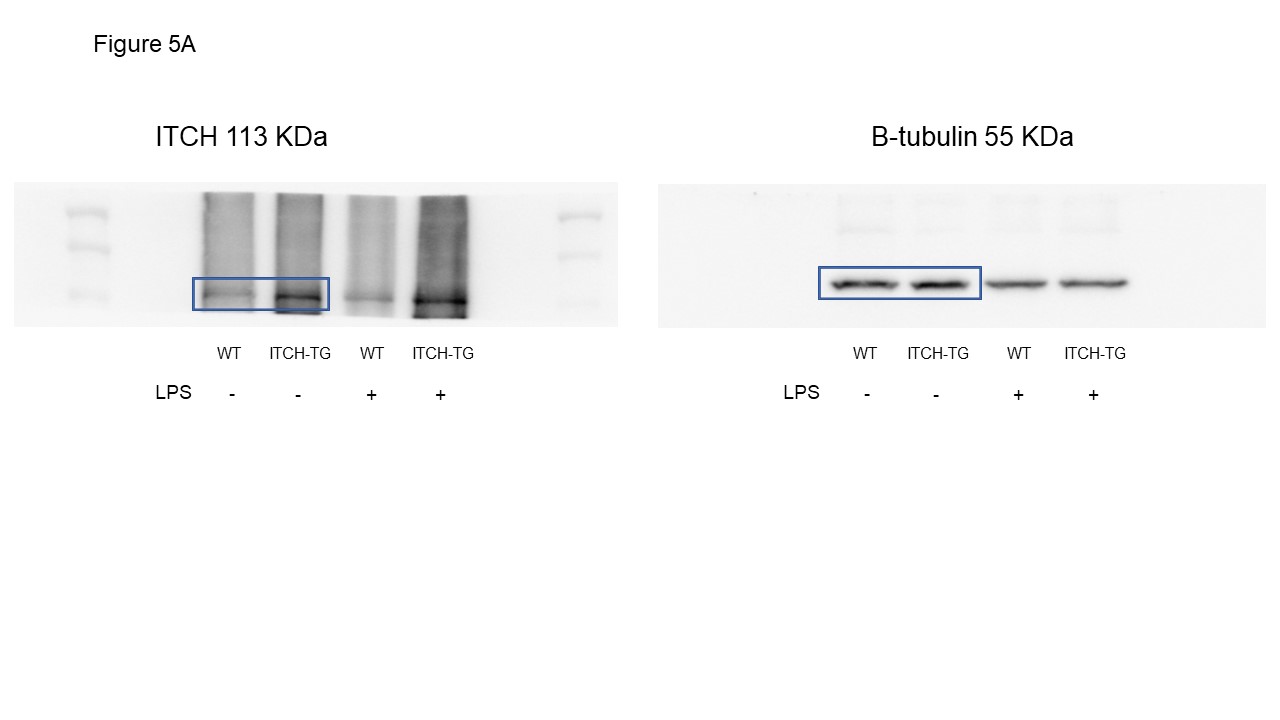


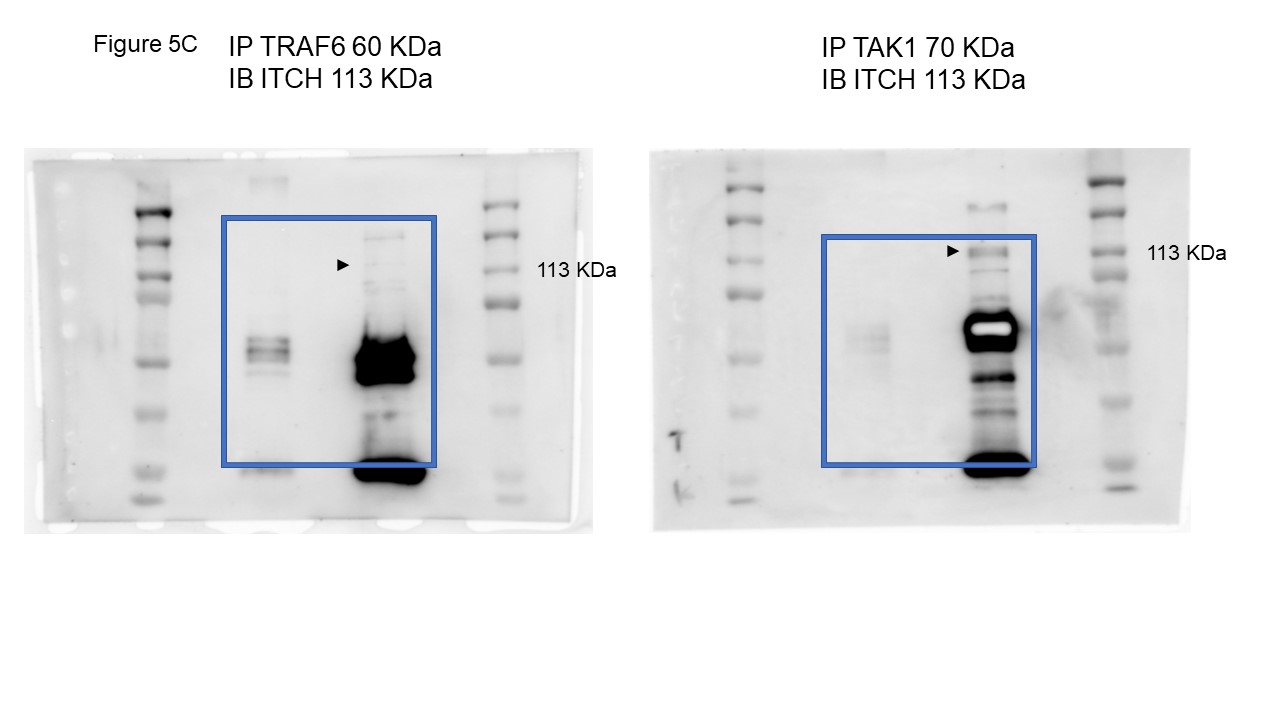


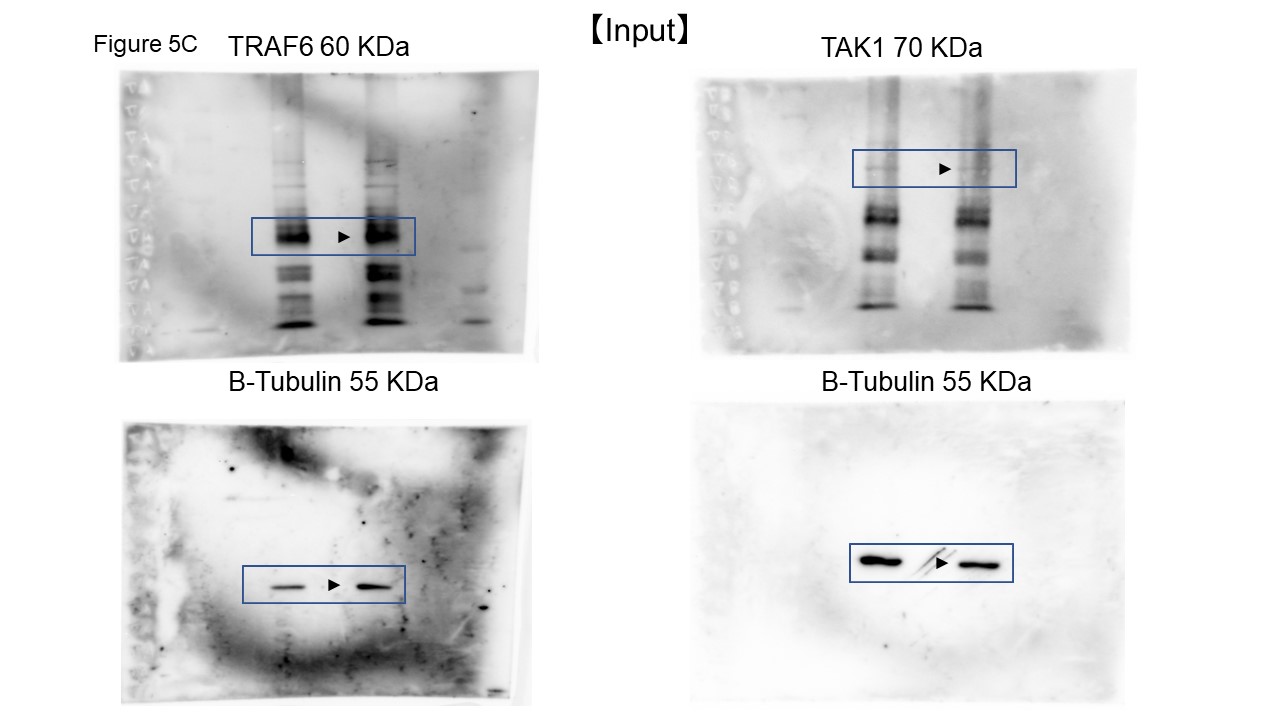


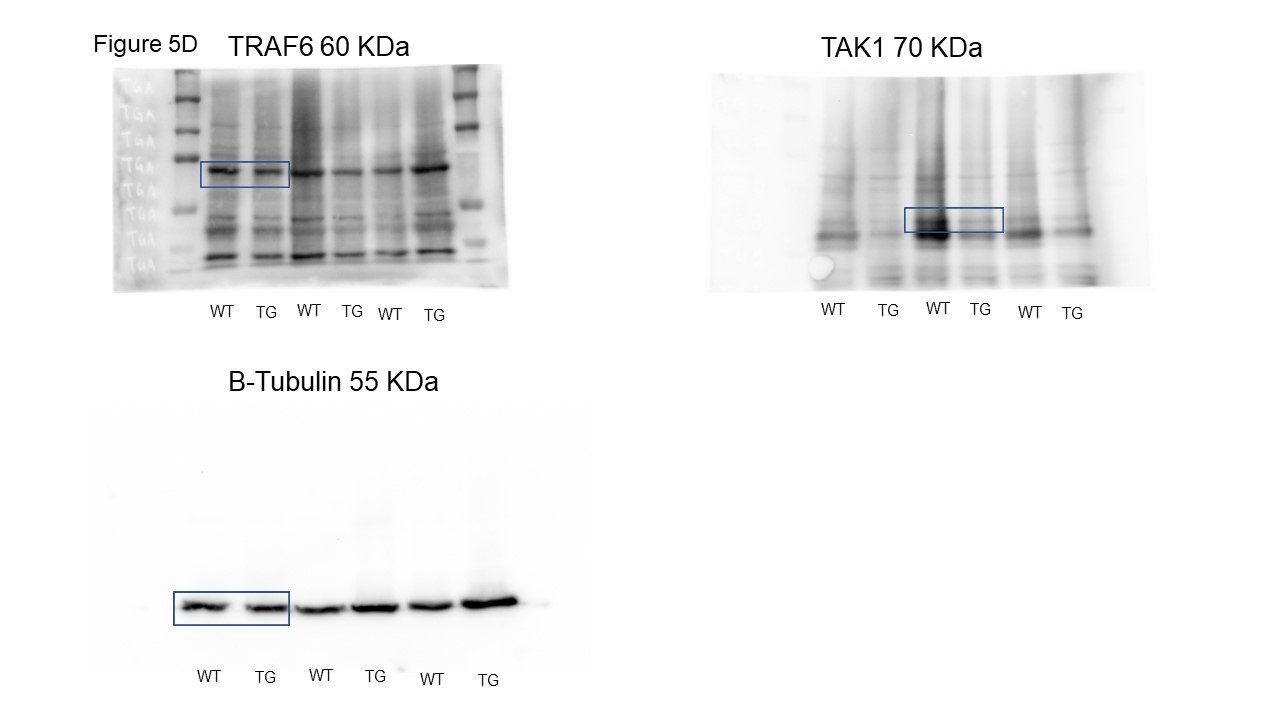


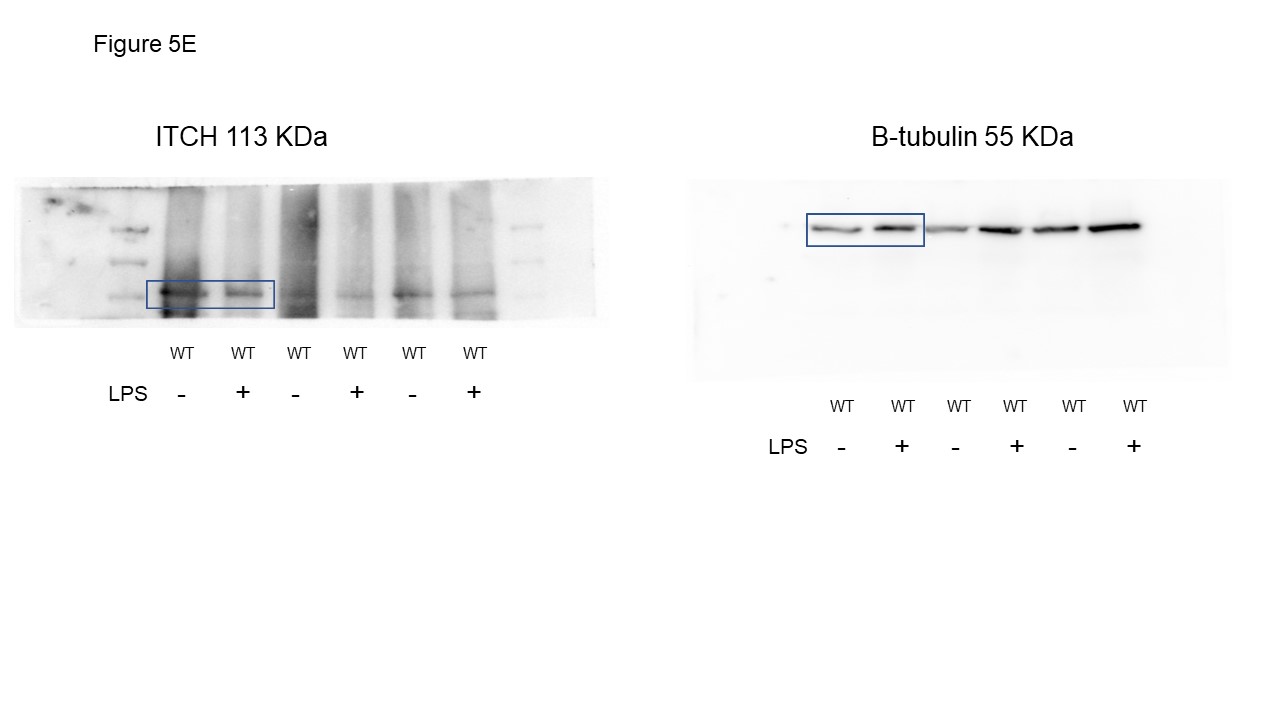


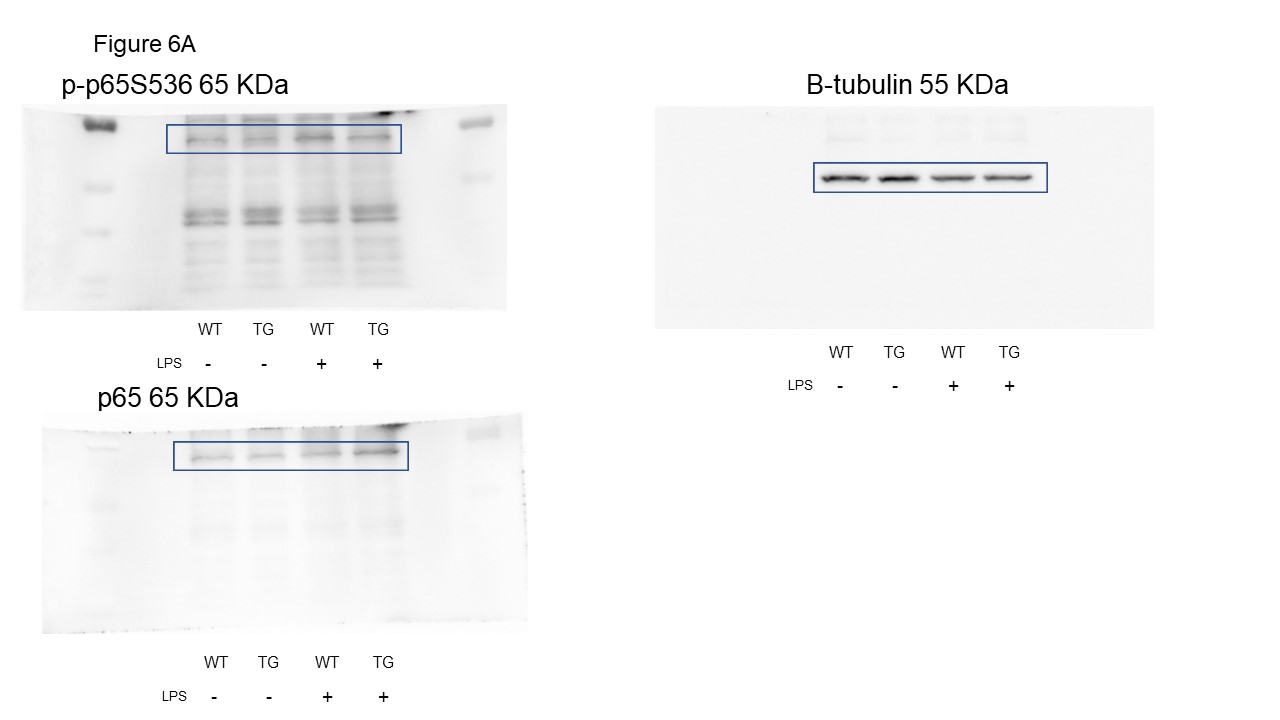

Supplement: Supplementary file 1 — Supplementary material [file mmc1.docx]
